# Supplementary material for: Joint embedding VQA model based on dynamic word vector
Source: PeerJ Comput Sci. 2021 Mar 3;7:e353. doi: 10.7717/peerj-cs.353 (PMC7959642; doi:10.7717/peerj-cs.353)
Supplement: Supplemental Information 1 [file peerj-cs-07-353-s001.zip › Learning to Answer Questions From Image Using Convolutional Neural Network.pdf]

arXiv:1506.00333v2 [cs.CL] 13 Nov 2015

Learning to Answer Questions From Image Using Convolutional Neural Network

Lin Ma Zhengdong Lu Hang Li
Noah's Ark Lab, Huawei Technologies
forest.linma@gmail.com Lu.Zhengdong@huawei.com HangLi,HL@huawei.com

Abstract

In this paper, we propose to employ the convolutional neural network (CNN) for the image question answering (QA). Our proposed CNN provides an end-to-end framework with convolutional architectures for learning not only the image and question representations, but also their inter-modal interactions to produce the answer. More specifically, our model consists of three CNNs: one image CNN to encode the image content, one sentence CNN to compose the words of the question, and one multimodal convolution layer to learn their joint representation for the classification in the space of candidate answer words. We demonstrate the efficacy of our proposed model on the DAQUAR and COCO-QA datasets, which are two benchmark datasets for the image QA, with the performances significantly outperforming the state-of-the-art.

Introduction

Recently, the multimodal learning between image and language (Ma et al. 2015; Makamura et al. 2013; Xu et al. 2015b) has become an increasingly popular research area of artificial intelligence (AI). In particular, there have been rapid progresses on the tasks of bidirectional image and sentence retrieval (Frome et al. 2013; Socher et al. 2014; Klein et al. 2015; Karpathy, Joulin, and Li 2014; Ordonez, Kulkarni, and Berg 2011), and automatic image captioning (Chen and Zitnick 2014; Karpathy and Li 2014; Donahue et al. 2014; Fang et al. 2014; Kiros, Salakhutdinov, and Zemel 2014a; Kiros, Salakhutdinov, and Zemel 2014b; Klein et al. 2015; Mao et al. 2014a; Mao et al. 2014b; Vinyals et al. 2014; Xu et al. 2015a). In order to further advance the multimodal learning and push the boundary of AI research, a new "AI-complete" task, namely the visual question answering (VQA) (Antol et al. 2015) or image question answering (QA) (Malinowski and Fritz 2014a; Malinowski and Fritz 2014b; Malinowski and Fritz 2015; Malinowski, Rohrbach, and Fritz 2015; Ren, Kiros, and Zemel 2015), is recently proposed. Generally, it takes an image and a free-form, natural-language like question about the image as the input and produces an answer to the image and question.

Image QA differs with the other multimodal learning tasks between image and sentence, such as the automatic

Copyright © 2015, Association for the Advancement of Artificial Intelligence (www.aaai.org). All rights reserved.

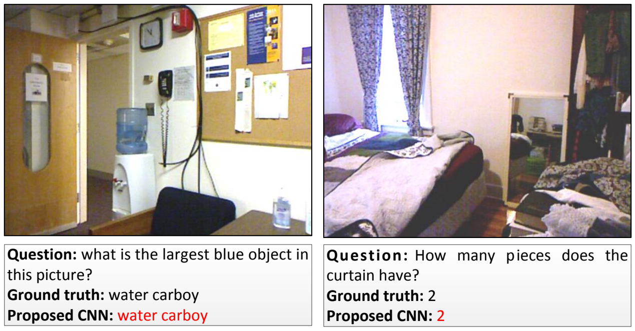

Figure 1: Samples of the image, the related question, and the ground truth answer, as well as the answer produced by our proposed CNN model.

image captioning. The answer produced by the image QA needs to be conditioned on both the image and question. As such, the image QA involves more interactions between image and language. As illustrated in Figure 1, the image contents are complicated, containing multiple different objects. The questions about the images are very specific, which requires a detailed understanding of the image content. For the question "`what is the largest blue object in this picture?`", we need not only identify the blue objects in the image but also compare their sizes to generate the correct answer. For the question "`how many pieces does the curtain have?`", we need to identify the object "`curtain`" in the non-salient region of the image and figure out its quantity.

A successful image QA model needs to be built upon good representations of the image and question. Recently, deep neural networks have been used to learn image and sentence representations. In particular, convolutional neural networks (CNNs) are extensively used to learn the image representation for image recognition (Simonyan and Zisserman 2014; Szegedy et al. 2015). CNNs (Hu et al. 2014; Kim 2014; Kalchbrenner, Grefenstette, and Blunsom 2014) also demonstrate their powerful abilities on the sentence representation for paraphrase, sentiment analysis, and so on. Moreover, deep neural networks (Mao et al. 2014a; Karpathy, Joulin, and Li 2014; Karpathy and Li 2014; Vinyals et al. 2014) are used to capture the relations between image and sentence for image captioning and retrieval.

However, for the image QA task, the ability of CNN has not been studied.

In this paper, we employ CNN to address the image QA problem. Our proposed CNN model, trained on a set of triplets consisting of (image, question, answer), can answer free-form, natural-language like questions about the image. Our main contributions are:

1. We propose an end-to-end CNN model for learning to answer questions about the image. Experimental results on public image QA datasets show that our proposed CNN model surpasses the state-of-the-art.
2. We employ convolutional architectures to encode the image content, represent the question, and learn the interactions between the image and question representations, which are jointly learned to produce the answer conditioning on the image and question.

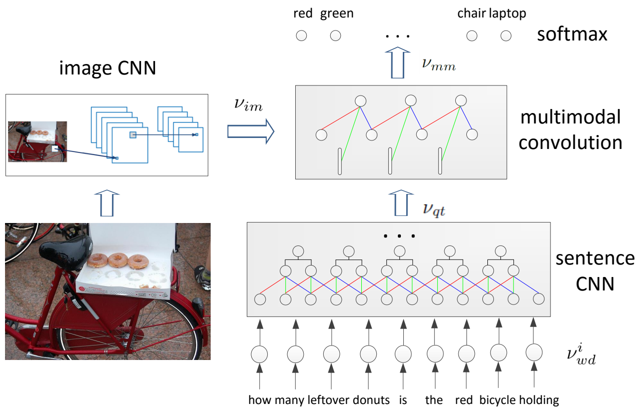

Figure 2: The proposed CNN model for image QA.

Related Work

Recently, the visual Turing test, an open domain task of question answering based on real-world images, has been proposed to resemble the famous Turing test. In (Gao et al. 2015) a human judge will be presented with an image, a question, and the answer to the question by the computational models or human annotators. Based on the answer, the human judge needs to determine whether the answer is given by a human (i.e. pass the test) or a machine (i.e. fail the test). Geman et al. (Geman et al. 2015) proposed to produce a stochastic sequence of binary questions from a given test image, where the answer to the question is limited to yes/no. Malinowski et al. (Malinowski and Fritz 2014b; Malinowski and Fritz 2015) further discussed the associated challenges and issues with regard to visual Turing test, such as the vision and language representations, the common sense knowledge, as well as the evaluation.

The image QA task, resembling the visual Turing test, is then proposed. Malinowski et al. (Malinowski and Fritz 2014a) proposed a multi-world approach that conducts the semantic parsing of question and segmentation of image to produce the answer. Deep neural networks are also employed for the image QA task, which is more related to our research work. The work by (Malinowski, Rohrbach, and Fritz 2015; Gao et al. 2015) formulates the image QA task as a generation problem. Malinowski et al.'s model (Malinowski, Rohrbach, and Fritz 2015), namely the Neural-Image-QA, feeds the image representation from CNN and the question into the long-short term memory (LSTM) to produce the answer. This model ignores the different characteristics of questions and answers. Compared with the questions, the answers tend to be short, such as one single word denoting the object category, color, number, and so on. The deep neural network in (Gao et al. 2015), inspired by the multimodal recurrent neural networks model (Mao et al. 2014b; Mao et al. 2014a), used two LSTMs for the representations of question and answer, respectively. In (Ren, Kiros, and Zemel 2015), the image QA task is formulated as a classification problem, and the so-called visual semantic embedding (VSE) model is proposed. LSTM is employed to jointly model the image and question by treating the image as an independent word, and appending it to the question at the beginning or ending position. As such, the joint representation of image and question is learned, which is further used for classification. However, simply treating the image as an individual word cannot help effectively exploit the complicated relations between the image and question. Thus, the accuracy of the answer prediction may not be ensured. In order to cope with these drawbacks, we proposed to employ an end-to-end convolutional architectures for the image QA to capture the complicated inter-modal relationships as well as the representations of image and question. Experimental results demonstrate that the convolutional architectures can can achieve better performance for the image QA task.

Proposed CNN for Image QA

For image QA, the problem is to predict the answer a given the question q and the related image I:

$$a = \underset{a \in \Omega}{\operatorname{argmax}}\ p(a|q, I; \theta), \quad (1)$$

where Ω is the set containing all the answers. θ denotes all the parameters for performing image QA. In order to make a reliable prediction of the answer, the question q and image I need to be adequately represented. Based on their representations, the relations between the two multimodal inputs are further learned to produce the answer. In this paper, the ability of CNN is exploited for not only modeling image and sentence individually, but also capturing the relations and interactions between them.

As illustrated in Figure 2, our proposed CNN framwork for image QA consists of three individual CNNs: one image CNN encoding the image content, one sentence CNN generating the question representation, one multimodal convolution layer fusing the image and question representations together and generate the joint representation. Finally, the joint representation is fed into a softmax layer to produce the answer. The three CNNs and softmax layer are fully coupled for our proposed end-to-end image QA framework, with all the parameters (three CNNs and softmax) jointly learned in an end-to-end fashion.

Image CNN

There are many research papers employing CNNs to generate image representations, which achieve the state-of-the-art performances on image recognition (Simonyan and Zisserman 2014; Szegedy et al. 2015). In this paper, we employ the work (Simonyan and Zisserman 2014) to encode the image content for our image QA model:

$$\nu_{im} = \sigma(\mathbf{w}_{im}(CNN_{im}(I)) + b_{im}), \tag{2}$$

where σ is a nonlinear activation function, such as Sigmoid and ReLU (Dahl, Sainath, and Hinton 2013). CNN_{im} takes the image as the input and outputs a fixed length vector as the image representation. In this paper, by chopping out the top softmax layer and the last ReLU layer of the CNN (Simonyan and Zisserman 2014), the output of the last fully-connected layer is deemed as the image representation, which is a fixed length vector with dimension as 4096. Note that $\mathbf{w}_{im}$ is a mapping matrix of the dimension $d \times 4096$, with d much smaller than 4096. On one hand, the dimension of the image representation is reduced from 4096 to d. As such, the total number of parameters for further fusing image and question, specifically the multimodal convolution process, is significantly reduced. Consequently, fewer samples are needed for adequately training our CNN model. On the other hand, the image representation is projected to a new space, with the nonlinear activation function σ increasing the nonlinear modeling property of the image CNN. Thus its capability for learning complicated representations is enhanced. As a result, the multimodal convolution layer (introduced in the following section) can better fuse the question and image representations together and further exploit their complicated relations and interactions to produce the answer.

Sentence CNN

In this paper, CNN is employed to model the question for image QA. As most convolution models (Lecun and Bengio 1995; Kalchbrenner, Grefenstette, and Blunsom 2014), we consider the convolution unit with a local "receptive field" and shared weights to capture the rich structures and composition properties between consecutive words. The sentence CNN for generating the question representation is illustrated in Figure 3. For a given question with each word represented as the word embedding (Mikolov et al. 2013), the sentence CNN with several layers of convolution and max-pooling is performed to generate the question representation ν_{qt}.

Convolution For a sequential input ν, the convolution unit for feature map of type-f on the ℓth layer is

$$\nu^i_{(\ell,f)} \stackrel{\text{def}}{=} \sigma(\mathbf{w}_{(\ell,f)}\vec{\nu}^i_{(\ell-1)} + b_{(\ell,f)}), \tag{3}$$

where $\mathbf{w}_{(\ell,f)}$ are the parameters for the f feature map on the ℓth layer, σ is the nonlinear activation function, and $\vec{\nu}^i_{(\ell-1)}$ denotes the segment of $(\ell-1)^{th}$ layer for the convolution at location i , which is defined as follows.

$$\vec{\nu}^i_{(\ell-1)} \stackrel{\text{def}}{=} \nu^i_{(\ell-1)} \parallel \nu^{i+1}_{(\ell-1)} \parallel \cdots \parallel \nu^{i+s_{rp}-1}_{(\ell-1)}, \tag{4}$$

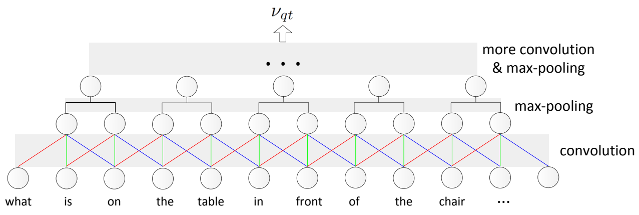

Figure 3: The sentence CNN for the question representation.

where s_{rp} defines the size of local "receptive field" for convolution. "$\parallel$" concatenates the s_{rp} vectors into a long vector. In this paper, s_{rp} is chosen as 3 for the convolution process. The parameters within the convolution unit are shared for the whole question with a window covering 3 semantic components sliding from the beginning to the end. The input of the first convolution layer for the sentence CNN is the word embeddings of the question:

$$\vec{\nu}^i_{(0)} \stackrel{\text{def}}{=} \nu^i_{wd} \parallel \nu^{i+1}_{wd} \parallel \cdots \parallel \nu^{i+s_{rp}-1}_{wd}, \tag{5}$$

where ν^i_{wd} is the word embedding of the ith word in the question.

Max-pooling With the convolution process, the sequential s_{rp} semantic components are composed to a higher semantic representation. However, these compositions may not be the meaningful representations, such as "`is on the`" of the question in Figure 3. The max-pooling process following each convolution process is performed:

$$\nu^i_{(\ell+1,f)} = \max(\nu^{2i}_{(\ell,f)}, \nu^{2i+1}_{(\ell,f)}). \tag{6}$$

Firstly, together with the stride as two, the max-pooling process shrinks half of the representation, which can quickly make the sentence representation. Most importantly, the max-pooling process can select the meaningful compositions while filter out the unreliable ones. As such, the meaningful composition "`of the chair`" is more likely to be pooled out, compared with the composition "`front of the`".

The convolution and max-pooling processes exploit and summarize the local relation signals between consecutive words. More layers of convolution and max-pooling can help to summarize the local interactions between words at larger scales and finally reach the whole representation of the question. In this paper, we employ three layers of convolution and max-pooling to generate the question representation ν_{qt}.

Multimodal Convolution Layer

The image representation ν_{im} and question representation ν_{qt} are obtained by the image and sentence CNNs, respectively. We design a new multimodal convolution layer on top of them, as shown in Figure 4, which fuses the multimodal inputs together to generate their joint representation for further answer prediction. The image representation is treated as an individual semantic component. Based on the image representation and the two consecutive semantic components from the question side, the mulitmodal convolution

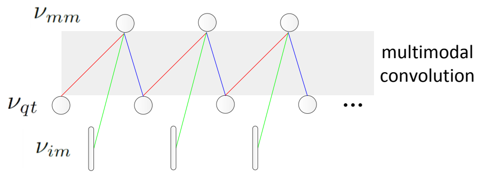

Figure 4: The multimodal convolution layer to fuse the image and question representations.

is performed, which is expected to capture the interactions and relations between the two multimodal inputs.

$$\vec{\nu}_{mm}^{in} \stackrel{\text{def}}{=} \nu_{qt}^{i} \parallel \nu_{im} \parallel \nu_{qt}^{i+1}, \tag{7}$$

$$\nu_{(mm,f)}^{i} \stackrel{\text{def}}{=} \sigma(\mathbf{w}_{(mm,f)}\vec{\nu}_{mm}^{in} + b_{(mm,f)}), \tag{8}$$

where $\vec{\nu}_{mm}^{in}$ is the input of the multimodal convolution unit. ν_{qt}^{i} is the segment of the question representation at location i. $\mathbf{w}_{(mm,f)}$ and $b_{(mm,f)}$ are the parameters for the type-f feature map of the multimodal convolution layer.

Alternatively, LSTM could be used to fuse the image and question representations, as in (Malinowski, Rohrbach, and Fritz 2015; Ren, Kiros, and Zemel 2015). For example, in the latter work, a bidirectional LSTM (Ren, Kiros, and Zemel 2015) is employed by appending the image representation to the beginning or ending position of the question. We argue that it is better to employ CNN than LSTM for the image QA task, due to the following reason, which has also been verified in the following experiment section. The relations between image and question are complicated. The image may interact with the high-level semantic representations composed from of a number of words, such as "`the red bicycle`" in Figure 2. However, LSTM cannot effectively capture such interactions. Treating the image representation as an individual word, the effect of image will vanish at each time step of LSTM in (Ren, Kiros, and Zemel 2015). As a result, the relations between the image and the high-level semantic representations of words may not be well exploited. In contrast, our CNN model can effectively deal with the problem. The sentence CNN first compose the question into a high-level semantic representations. The multimodal convolution process further fuse the semantic representations of image and question together and adequately exploit their interactions.

After the mutlimodal convolution layer, the multimodal representation ν_{mm} jointly modeling the image and question is obtained. ν_{mm} is then fed into a softmax layer as shown in Figure 2, which produces the answer to the given image and question pair.

Experiments

In this section, we firstly introduce the configurations of our CNN model for image QA and how we train the proposed CNN model. Afterwards, the public image QA datasets and evaluation measurements are introduced. Finally, the experimental results are presented and analyzed.

Configurations and Training

Three layers of convolution and max-pooling are employed for the sentence CNN. The numbers of the feature maps for the three convolution layers are 300, 400, and 400, respectively. The sentence CNN is designed on a fixed architecture, which needs to be set to accommodate the maximum length of the questions. In this paper, the maximum length of the question is chosen as 38. The word embeddings are obtained by the skip-gram model (Mikolov et al. 2013) with the dimension as 50. We use the VGG (Simonyan and Zisserman 2014) network as the image CNN. The dimension of ν_{im} is set as 400. The multimodal CNN takes the image and sentence representations as the input and generate the joint representation with the number of feature maps as 400.

The proposed CNN model is trained with stochastic gradient descent with mini batches of 100 for optimization, where the negative log likelihood is chosen as the loss. During the training process, all the parameters are tuned, including the parameters of nonlinear image mapping, image CNN, sentence CNN, multimodal convolution layer, and softmax layer. Moreover, the word embeddings are also fine-tuned. In order to prevent overfitting, dropout (with probability 0.1) is used.

Image QA Datasets

We test and compare our proposed CNN model on the public image QA databases, specifically the DAQUAR (Malinowski and Fritz 2014a) and COCO-QA (Ren, Kiros, and Zemel 2015) datasets.

DAQUAR-All (Malinowski and Fritz 2014a) This dataset consists of 6,795 training and 5,673 testing samples, which are generated from 795 and 654 images, respectively. The images are from all the 894 object categories. There are mainly three types of questions in this dataset, specifically the object type, object color, and number of objects. The answer may be a single word or multiple words.

DAQUAR-Reduced (Malinowski and Fritz 2014a) This dataset is a reduced version of DAQUAR-All, comprising 3,876 training and 297 testing samples. The images are constrained to 37 object categories. Only 25 images are used for the testing sample generation. Same as the DAQUAR-All dataset, the answer may be a single word or multiple words.

COCO-QA (Ren, Kiros, and Zemel 2015) This dataset consists of 79,100 training and 39,171 testing samples, which are generated from about 8,000 and 4,000 images, respectively. There are four types of questions, specifically the object, number, color, and location. The answers are all single-word.

Evaluation Measurements

One straightforward way for evaluating image QA is to utilize accuracy, which measures the proportion of the correctly answered testing questions to the total testing questions. Besides accuracy, Wu-Palmer similarity (WUPS) (Wu and Palmer 1994; Malinowski and Fritz 2014a) is also used to measure the performances of different models on the image QA task. WUPS calculates the similarity between two words based on their common subsequence in a taxonomy tree.

Table 1: Image QA performances on DAQUAR-All.

	Accuracy	WUPS @0.9	WUPS @0.0
Multi-World Approach (Malinowski and Fritz 2014a)	7.86	11.86	38.79
Human Answers (Malinowski, Rohrbach, and Fritz 2015)	50.20	50.82	67.27
Human Answers without image (Malinowski, Rohrbach, and Fritz 2015)	11.99	16.82	33.57
Neural-Image-QA (Malinowski, Rohrbach, and Fritz 2015)			
-multiple words	17.49	23.28	57.76
-single word	19.43	25.28	62.00
Language Approach			
-multiple words	17.06	22.30	56.53
-single word	17.15	22.80	58.42
Proposed CNN			
-multiple words	**21.47**	**27.15**	**59.44**
-single word	**24.49**	**30.47**	**66.08**

A threshold parameter is required for the calculation of WUPS. Same as the previous work (Ren, Kiros, and Zemel 2015; Malinowski and Fritz 2014a; Malinowski, Rohrbach, and Fritz 2015), the threshold parameters 0.0 and 0.9 are used for the measurements WUPS@0.0 and WUPS@0.9, respectively.

Experimental Results and Analysis

Competitor Models We compare our models with recently developed models for the image QA task, specifically the multi-world approach (Malinowski and Fritz 2014a), the VSE model (Ren, Kiros, and Zemel 2015), and the Neural-Image-QA approach (Malinowski, Rohrbach, and Fritz 2015).

Performances on Image QA The performances of our proposed CNN model on the DAQUAR-All, DAQUAR-Reduced, and COCO-QA datasets are illustrated in Table 1, 2, and 3, respectively. For DAQUAR-All and DAQUAR-Reduced datasets with multiple words as the answer to the question, we treat the answer comprising multiple words as an individual class for training and testing.

For the DAQUAR-All dataset, we evaluate the performances of different image QA models on the full set ("multiple words"). The answer to the image and question pair may be a single word or multiple words. Same as the work (Malinowski, Rohrbach, and Fritz 2015), a subset containing the samples with only a single word as the answer is created and employed for comparison ("single word"). Our proposed CNN model significantly outperforms the multi-world approach and Neural-Image-QA in terms of accuracy, WUPS@0.0, and WUPS@0.9. Specifically, our proposed CNN model achieves over 20% improvement compared to Neural-Image-QA in terms of accuracy on both "multiple words" and "single word". The results, shown in Table. 1, demonstrate that our CNN model can more accurately model the image and question as well as their interactions, thus yields better performances for the image QA task. Moreover, the language approach (Malinowski, Rohrbach, and Fritz 2015), which only resorts to the question performs inferiorly to the approaches that jointly model the image and question. The image component is thus of great help to the image QA task. One can also see that the performances on "multiple words" are generally inferior to those on "single word".

Table 2: Image QA performances on DAQUAR-Reduced.

	Accuracy	WUPS @0.9	WUPS @0.0
Multi-World Approach (Malinowski and Fritz 2014a)	12.73	18.10	51.47
Neural-Image-QA (Malinowski, Rohrbach, and Fritz 2015)			
-multiple words	29.27	36.50	79.47
-single word	34.68	40.76	79.54
Language Approach			
-multiple words	32.32	38.39	80.05
-single word	31.65	38.35	80.08
VSE (Ren, Kiros, and Zemel 2015)			
-single word			
GUESS	18.24	29.65	77.59
BOW	32.67	43.19	81.30
LSTM	32.73	43.50	81.62
IMG+BOW	34.17	44.99	81.48
VIS+LSTM	34.41	46.05	82.23
2-VIS+BLSTM	35.78	46.83	82.14
Proposed CNN			
-multiple words	**38.38**	**43.43**	**80.63**
-single word	**42.76**	**47.58**	**82.60**

For the DAQUAR-Reduced dataset, besides the Neural-Image-QA approach, the VSE model is also compared on "single word". Moreover, some of the methods introduced in (Ren, Kiros, and Zemel 2015) are also reported and compared. GUESS is the model which randomly outputs the answer according to the question type. BOW treats each word of the question equally and sums all the word vectors to predict the answer by logistic regression. LSTM is performed only on the question without considering the image, which is similar to the language approach (Malinowski, Rohrbach, and Fritz 2015). IMG+BOW performs the multinomial logistic regression based on the image feature and a BOW vector obtained by summing all the word vectors of the question. VIS+LSTM and 2-VIS+BLSTM are two versions of the VSE model. VIS+LSTM has only a single LSTM to encode the image and question in one direction, while 2-VIS+BLSTM uses a bidirectional LSTM to encode the image and question along with both directions to fully exploit the interactions between image and each word of the question. It can be observed that 2-VIS+BLSTM outperforms VIS+LSTM with a big margin. The same observation can also be found on the COCO-QA dataset, as shown in Table 3, demonstrating that the bidirectional LSTM can more accurately model the interactions between image and question than the single LSTM. Our proposed CNN model significantly outperforms the competitor models. More specifically, for the case of "single word", our proposed CNN achieves nearly 20% improvement in terms of accuracy over the best competitor model 2-VIS+BLSTM.

For the COCO-QA dataset, IMG+BOW outperforms VIS+LSTM and 2-VIS+BLSTM, demonstrating that the

Table 3: Image QA performances on COCO-QA.

	Accuracy	WUPS @0.9	WUPS @0.0
VSE (Ren, Kiros, and Zemel 2015)			
GUESS	6.65	17.42	73.44
BOW	37.52	48.54	82.78
LSTM	36.76	47.58	82.34
IMG	43.02	58.64	85.85
IMG+BOW	55.92	66.78	88.99
VIS+LSTM	53.31	63.91	88.25
2-VIS+BLSTM	55.09	65.34	88.64
FULL	57.84	67.90	89.52
Proposed CNN without multimodal convolution layer	56.77	66.76	88.94
Proposed CNN without image representation	37.84	48.70	82.92
Proposed CNN	**58.40**	**68.50**	**89.67**

simple multinomial logistic regression of IMG+BOW can better model the interactions between image and question, compared with the LSTMs of VIS+LSTM and 2-VIS+BLSTM. By averaging VIS+LSTM, 2-VIS+BLSTM, and IMG+BOW, the FULL model is developed, which summarizes the interactions between image and question from different perspectives thus yields a much better performance. As shown in Table 3, our proposed CNN model outperforms all the competitor models in terms of all the three evaluation measurements, even the FULL model. The reason may be that the image representation is of highly semantic meaning, which should interact with the high semantic components of the question. Our CNN model firstly uses the convolutional architectures to compose the words to highly semantic representations. Afterwards, we let the image meet the composed highly semantic representations and use convolutional architectures to exploit their relations and interactions for the answer prediction. As such, Our CNN model can well model the relations between image and question, and thus obtain the best performances.

Influence of Multimodal Convolution Layer The image and question needs to be considered together for the image QA. The multimodal convolution layer in our proposed CNN model not only fuses the image and question representations together but also learns the interactions and relations between the two multimodal inputs for further question prediction. The effect of the multimodal convolution layer is examined as follows. The image and question representations are simply concatenated together as the input of the softmax layer for the answer prediction. We train the network in the same manner as the proposed CNN model. The results are provided in Table 3. Firstly, it can be observed that without the multimodal convolution layer, the performance on the image QA has dropped. Comparing to the simple concatenation process fusing the image and question representations, our proposed multimodal convolution layer can well exploit the complicated relationships between image and question representations. Thus a better performance for the answer prediction is achieved. Secondly, the approach without multimodal convolution layer outperforms the IMG+BOW, VIS+LSTM and 2-VIS+BLSTM, in terms of accuracy. The better performance is mainly attributed to the composition ability of the sentence CNN. Even with the simple concatenation process, the image representation and composed question representation can be fuse together for a better image QA model.

Influence of Image CNN and Effectiveness of Sentence CNN As can be observed in Table 1, without the image content, the accuracy of human answering the question drops from 50% to 12%. Therefore, the image content is critical to the image QA task. Same as the work (Malinowski, Rohrbach, and Fritz 2015; Ren, Kiros, and Zemel 2015), we only use the question representation obtained from the sentence CNN to predict the answer. The results are listed in Table 3. Firstly, without the use of image representation, the performance of our proposed CNN significantly drops, which again demonstrates the importance of image component to the image QA. Secondly, the model only consisting of the sentence CNN performs better than LSTM and BOW for the image QA. It indicates that the sentence CNN is more effective to generate the question representation for image QA, compared with LSTM and BOW. Recall that the model without multimodal convolution layers outperforms IMG+BOW, VIS+LSTM, and 2-VIS+BLSTM, as explained above. By incorporating the image representation, the better modeling ability of our sentence CNN is demonstrated.

Moreover, we examine the language modeling ability of the sentence CNN as follows. The words of the test questions are randomly reshuffled. Then the reformulated questions are sent to the sentence CNN to check whether the sentence CNN can still generate reliable question representations and make accurate answer predictions. For randomly reshuffled questions, the results on COCO-QA dataset are 40.74, 53.06, and 80.41 for the accuracy, WUPS@0.9, and WUPS@0.0, respectively, which are significantly inferior to that of natural-language like questions. The result indicates that the sentence CNN possesses the ability of modeling natural questions. The sentence CNN uses the convolution process to compose and summarize the neighboring words. And the reliable ones with higher semantic meanings will be pooled and composed further to reach the final sentence representation. As such, the sentence CNN can compose the natural-language like questions to reliable high semantic representations.

Conclusion

In this paper, we proposed one CNN model to address the image QA problem. The proposed CNN model relies on convolutional architectures to generate the image representation, compose consecutive words to the question representation, and learn the interactions and relations between the image and question for the answer prediction. Experimental results on public image QA datasets demonstrate the superiority of our proposed model over the state-of-the-art methods.

Acknowledgement

The work is partially supported by China National 973 project 2014CB340301. The authors are grateful to Baotian Hu and Zhenguo Li for their insightful discussions and comments.

References

[Antol et al. 2015] Antol, S.; Agrawal, A.; Lu, J.; Mitchell, M.; Batra, D.; Zitnick, C. L.; and Parikh, D. 2015. VQA: visual question answering. *arXiv 1505.00468*.

[Chen and Zitnick 2014] Chen, X., and Zitnick, C. L. 2014. Learning a recurrent visual representation for image caption generation. *arXiv 1411.5654*.

[Dahl, Sainath, and Hinton 2013] Dahl, G. E.; Sainath, T. N.; and Hinton, G. E. 2013. Improving deep neural networks for LVCSR using rectified linear units and dropout. In *ICASSP*.

[Donahue et al. 2014] Donahue, J.; Hendricks, L. A.; Guadarrama, S.; Rohrbach, M.; Venugopalan, S.; Saenko, K.; and Darrell, T. 2014. Long-term recurrent convolutional networks for visual recognition and description. *arXiv 1411.4389*.

[Fang et al. 2014] Fang, H.; Gupta, S.; Iandola, F. N.; Srivastava, R.; Deng, L.; Dollár, P.; Gao, J.; He, X.; Mitchell, M.; Platt, J. C.; Zitnick, C. L.; and Zweig, G. 2014. From captions to visual concepts and back. *arXiv 1411.4952*.

[Frome et al. 2013] Frome, A.; Corrado, G.; Shlens, J.; Bengio, S.; Dean, J.; Ranzato, M.; and Mikolov, T. 2013. Devise: A deep visual-semantic embedding model. In *NIPS*.

[Gao et al. 2015] Gao, H.; Mao, J.; Zhou, J.; Huang, Z.; Wang, L.; and Xu, W. 2015. Are you talking to a machine? dataset and methods for multilingual image question answering. *arXiv 1505.05612*.

[Geman et al. 2015] Geman, D.; Geman, S.; Hallonquist, N.; and Younes, L. 2015. Visual turing test for computer vision systems. In *PNAS*.

[Hu et al. 2014] Hu, B.; Lu, Z.; Li, H.; and Chen, Q. 2014. Convolutional neural network architectures for matching natural language sentences. In *NIPS*.

[Kalchbrenner, Grefenstette, and Blunsom 2014] Kalchbrenner, N.; Grefenstette, E.; and Blunsom, P. 2014. A convolutional neural network for modelling sentences. In *ACL*.

[Karpathy and Li 2014] Karpathy, A., and Li, F.-F. 2014. Deep visual-semantic alignments for generating image descriptions. *arXiv 1412.2306*.

[Karpathy, Joulin, and Li 2014] Karpathy, A.; Joulin, A.; and Li, F.-F. 2014. Deep fragment embeddings for bidirectional image sentence mapping. In *NIPS*.

[Kim 2014] Kim, Y. 2014. Convolutional neural networks for sentence classification. In *EMNLP*.

[Kiros, Salakhutdinov, and Zemel 2014a] Kiros, R.; Salakhutdinov, R.; and Zemel, R. 2014a. Multimodal neural language models. In *ICML*.

[Kiros, Salakhutdinov, and Zemel 2014b] Kiros, R.; Salakhutdinov, R.; and Zemel, R. S. 2014b. Unifying visual-semantic embeddings with multimodal neural language models. *arXiv 1411.2539*.

[Klein et al. 2015] Klein, B.; Lev, G.; Sadeh, G.; and Wolf, L. 2015. Fisher vectors derived from hybrid gaussian-laplacian mixture models for image annotation. In *CVPR*.

[Lecun and Bengio 1995] Lecun, Y., and Bengio, Y. 1995. Convolutional networks for images, speech and time series. *The Handbook of Brain Theory and Neural Networks*.

[Ma et al. 2015] Ma, L.; Lu, Z.; Shang, L.; and Li, H. 2015. Multimodal convolutional neural networks for matching image and sentence. In *ICCV*.

[Makamura et al. 2013] Makamura, T.; Nagai, T.; Funakoshi, K.; Nagasaka, S.; Taniguchi, T.; and Iwahashi, N. 2013. Mutual learning of an object concept and language model based on mlda and npylm. In *IROS*.

[Malinowski and Fritz 2014a] Malinowski, M., and Fritz, M. 2014a. A multi-world approach to question answering about real-world scenes based on uncertain input. In *NIPS*.

[Malinowski and Fritz 2014b] Malinowski, M., and Fritz, M. 2014b. Towards a visual turing challenge. *arXiv 1410.8027*.

[Malinowski and Fritz 2015] Malinowski, M., and Fritz, M. 2015. Hard to cheat: A turing test based on answering questions about images. *arXiv 1501.03302*.

[Malinowski, Rohrbach, and Fritz 2015] Malinowski, M.; Rohrbach, M.; and Fritz, M. 2015. Ask your neurons: A neural-based approach to answering questions about images. *arXiv 1505.01121*.

[Mao et al. 2014a] Mao, J.; Xu, W.; Yang, Y.; Wang, J.; and Yuille, A. L. 2014a. Deep captioning with multimodal recurrent neural networks (m-rnn). *arXiv 1412.6632*.

[Mao et al. 2014b] Mao, J.; Xu, W.; Yang, Y.; Wang, J.; and Yuille, A. L. 2014b. Explain images with multimodal recurrent neural networks. *arXiv 1410.1090*.

[Mikolov et al. 2013] Mikolov, T.; Chen, K.; Corrado, G.; and Dean, J. 2013. Efficient estimation of word representations in vector space. *arXiv 1301.3781*.

[Ordonez, Kulkarni, and Berg 2011] Ordonez, V.; Kulkarni, G.; and Berg, T. L. 2011. Im2text: Describing images using 1 million captioned photographs. In *NIPS*.

[Ren, Kiros, and Zemel 2015] Ren, M.; Kiros, R.; and Zemel, R. S. 2015. Exploring models and data for image question answering. *arXiv 1505.02074*.

[Simonyan and Zisserman 2014] Simonyan, K., and Zisserman, A. 2014. Very deep convolutional networks for large-scale image recognition. *arXiv 1409.1556*.

[Socher et al. 2014] Socher, R.; Karpathy, A.; Le, Q. V.; Manning, C. D.; and Ng, A. Y. 2014. Grounded compositional semantics for finding and describing images with sentences. In *TACL*.

[Szegedy et al. 2015] Szegedy, C.; Liu, W.; Jia, Y.; Sermanet, P.; Reed, S.; Anguelov, D.; Erhan, D.; Vanhoucke, V.; and Rabinovich, A. 2015. Going deeper with convolutions. In *CVPR*.

[Vinyals et al. 2014] Vinyals, O.; Toshev, A.; Bengio, S.; and

Erhan, D. 2014. Show and tell: A neural image caption generator. *arXiv 1411.4555*.

[Wu and Palmer 1994] Wu, Z., and Palmer, M. S. 1994. Verb semantics and lexical selection. In *ACL*.

[Xu et al. 2015a] Xu, K.; Ba, J.; Kiros, R.; Cho, K.; Courville, A.; Salakhutdinov, R.; Zemel, R.; and Bengio, Y. 2015a. Show, attend and tell: Neural image caption generation with visual attention. *arXiv 1502.03044*.

[Xu et al. 2015b] Xu, R.; Xiong, C.; Chen, W.; and Corso, J. 2015b. Jointly modeling deep video and compositional text to bridge vision and language in a unified framework. In *AAAI*.
