# Supplementary material for: Joint embedding VQA model based on dynamic word vector
Source: PeerJ Comput Sci. 2021 Mar 3;7:e353. doi: 10.7717/peerj-cs.353 (PMC7959642; doi:10.7717/peerj-cs.353)
Supplement: Supplemental Information 1 [file peerj-cs-07-353-s001.zip › Deep modular co-attention networks for visual question answering.pdf]

This CVPR paper is the Open Access version, provided by the Computer Vision Foundation.
Except for this watermark, it is identical to the accepted version;
the final published version of the proceedings is available on IEEE Xplore.

Deep Modular Co-Attention Networks for Visual Question Answering

Zhou Yu[1] Jun Yu[1]* Yuhao Cui[1] Dacheng Tao[2] Qi Tian[3]
[1]Key Laboratory of Complex Systems Modeling and Simulation,
School of Computer Science and Technology, Hangzhou Dianzi University, China.
[2]UBTECH Sydney AI Centre, School of Computer Science, FEIT, University of Sydney, Australia
[3]Noah's Ark Lab, Huawei, China
{yuz, yujun, cuiyh}@hdu.edu.cn, dacheng.tao@sydney.edu.au, tian.qi1@huawei.com

Abstract

Visual Question Answering (VQA) requires a fine-grained and simultaneous understanding of both the visual content of images and the textual content of questions. Therefore, designing an effective 'co-attention' model to associate key words in questions with key objects in images is central to VQA performance. So far, most successful attempts at co-attention learning have been achieved by using shallow models, and deep co-attention models show little improvement over their shallow counterparts. In this paper, we propose a deep Modular Co-Attention Network (MCAN) that consists of Modular Co-Attention (MCA) layers cascaded in depth. Each MCA layer models the self-attention of questions and images, as well as the question-guided-attention of images jointly using a modular composition of two basic attention units. We quantitatively and qualitatively evaluate MCAN on the benchmark VQA-v2 dataset and conduct extensive ablation studies to explore the reasons behind MCAN's effectiveness. Experimental results demonstrate that MCAN significantly outperforms the previous state-of-the-art. Our best single model delivers 70.63% overall accuracy on the test-dev set.

1. Introduction

Multimodal learning to bridge vision and language has gained broad interest from both the computer vision and natural language processing communities. Significant progress has been made in many vision-language tasks, including image-text matching [23, 14], visual captioning [9, 30, 1], visual grounding [10, 34] and visual question answering (VQA) [2, 21, 14, 36]. Compared to other multimodal learning tasks, VQA is a more challenging task that requires fine-grained semantic understanding of both

*Jun Yu is the corresponding author

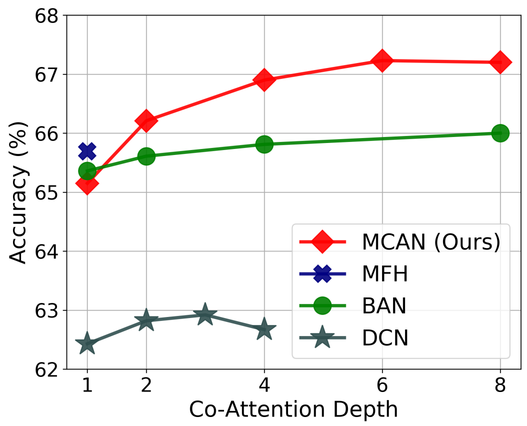

Figure 1: Accuracies *vs.* co-attention depth on VQA-v2 *val* split. We list most of the state-of-the-art approaches with (deep) co-attention models. Except for DCN [24] which uses the convolutional visual features and thus leads to inferior performance, all the compared methods (*i.e.*, MCAN, BAN [14] and MFH [33]) use the same bottom-up attention visual features to represent images [1].

the image and the question, together with visual reasoning to predict an accurate answer.

The attention mechanism is a recent advance in deep neural networks, that has successfully been applied to the unimodal tasks (*e.g.*, vision [22], language [4], and speech [8]), as well as the aforementioned multimodal tasks. The idea of learning visual attention on image regions from the input question in VQA was first proposed by [27, 7], and it becomes a de-facto component of almost all VQA approaches [10, 16, 1]. Along with visual attention, learning textual attention on the question key words is also very important. Recent works have shown that simultaneously learning co-attention for the visual and textual modalities can benefit the fine-grained representation of the image and question, leading to more accurate prediction [20, 33]. However, these co-attention models learn the coarse

interactions of multimodal instances, and the learned co-attention cannot infer the correlation between each image region and each question word. This results in a significant limitation of these co-attention models.

To overcome the problem of insufficient multimodal interactions, two dense co-attention models BAN [14] and DCN [24] have been proposed to model dense interactions between any image region and any question word. The dense co-attention mechanism facilitates the understanding of image-question relationship to correctly answer questions. Interestingly, both of these dense co-attention models can be cascaded in depth, form deep co-attention models that support more complex visual reasoning, thereby potentially improving VQA performance. However, these deep models shows little improvement over their corresponding shallow counterparts or the coarse co-attention model MFH [33] (see Figure 1). We think the bottleneck in these deep co-attention models is a deficiency of simultaneously modeling dense self-attention within each modality (*i.e.*, word-to-word relationship for questions, and region-to-region relationship for images).

Inspired by the Transformer model in machine translation [29], here we design two general attention units: a self-attention (SA) unit that can model the dense intra-modal interactions (word-to-word or region-to-region); and a guided-attention (GA) unit to model the dense inter-modal interactions (word-to-region). After that, by modular composition of the SA and GA units, we obtain different Modular Co-Attention (MCA) layers that can be cascaded in depth. Finally, we propose a deep Modular Co-Attention Network (MCAN) which consists of cascaded MCA layers. Results in Figure 1 shows that a deep MCAN model significantly outperforms existing state-of-the-art co-attention models on the benchmark VQA-v2 dataset [11], which verifies the synergy of self-attention and guided-attention in co-attention learning, and also highlights the potential of deep reasoning. Furthermore, we find that modeling self-attention for image regions can greatly improve the object counting performance, which is challenging for VQA.

2. Related Work

We briefly review previous studies on VQA, especially those studies that introduce co-attention models.

Visual Question Answering (VQA). VQA has been of increasing interest over the last few years. The multimodal fusion of global features are the most straightforward VQA solutions. The image and question are first represented as global features and then fused by a multimodal fusion model to predict the answer [37]. Some approaches introduce a more complex model to learn better question representations with LSTM networks [2], or a better multimodal fusion model with residual networks [15].

One limitation of the aforementioned multimodal fusion models is that the global feature representation of an image may lose critical information to correctly answer the questions about local image regions (*e.g.*, "what is in the woman's left hand"). Therefore, recent approaches have introduced the visual attention mechanism into VQA by adaptively learning the attended image features for a given question, and then performing multimodal feature fusion to obtain the accurate prediction. Chen *et al.* proposed a question-guided attention map that projected the question embeddings into the visual space and formulated a configurable convolutional kernel to search the image attention region [7]. Yang *et al.* proposed a stacked attention network to learn the attention iteratively [31]. Fukui *et al.* [10], Kim *et al.* [16], Yu *et al.* [32, 33] and Ben *et al.* [6] exploited different multimodal bilinear pooling methods to integrate the visual features from the image's spatial grids with the textual features from the questions to predict the attention. Anderson *et al.* introduced a bottom-up and top-down attention mechanism to learn the attention on candidate objects rather than spatial grids [1].

Co-Attention Models. Beyond understanding the visual contents of the image, VQA also requires to fully understand the semantics of the natural language question. Therefore, it is necessary to learn the textual attention for the question and the visual attention for the image simultaneously. Lu *et al.* proposed a co-attention learning framework to alternately learn the image attention and question attention [20]. Yu *et al.* reduced the co-attention method into two steps, self-attention for a question embedding and the question-conditioned attention for a visual embedding [33]. Nam *et al.* proposed a multi-stage co-attention learning model to refine the attentions based on memory of previous attentions [23]. However, these co-attention models learn separate attention distributions for each modality (image or question), and neglect the dense interaction between each question word and each image region. This become a bottleneck for understanding fine-grained relationships of multimodal features. To address this issue, dense co-attention models have been proposed, which establish the complete interaction between each question word and each image region [24, 14]. Compared to the previous co-attention models with coarse interactions, the dense co-attention models deliver significantly better VQA performance.

3. Modular Co-Attention Layer

Before presenting the Modular Co-Attention Network, we first introduce its basic component, the Modular Co-Attention (MCA) layer. The MCA layer is a modular composition of the two basic attention units, *i.e.*, the self-attention (SA) unit and the guided-attention (GA) unit, inspired by the *scaled dot-product attention* proposed in

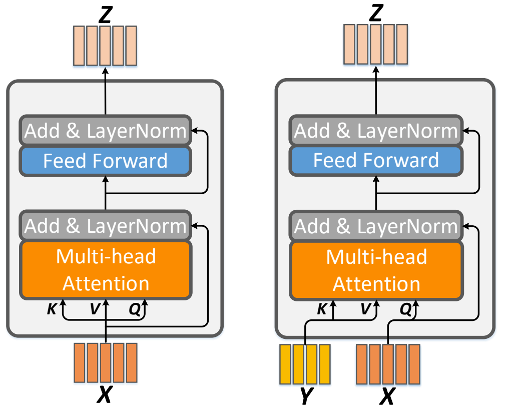

(a) Self-Attention (SA) (b) Guided-Attention (GA)

Figure 2: Two basic attention units with multi-head attention for different types of inputs. SA takes one group of input features X and output the attended features Z for X; GA takes two groups of input features X and Y and output the attended features Z for X guided by Y.

[29]. Using different combinations, we obtain three MCA variants with different motivations.

3.1. Self-Attention and Guided-Attention Units

The input of scaled dot-product attention consists of queries and keys of dimension d_{key}, and values of dimension d_{value}. For simplicity, d_{key} and d_{value} are usually set to the same number d. We calculate the dot products of the query with all keys, divide each by $\sqrt{d}$ and apply a softmax function to obtain the attention weights on the values. Given a query $q \in \mathbb{R}^{1\times d}$, n key-value pairs (packed into a key matrix $K \in \mathbb{R}^{n\times d}$ and a value matrix $V \in \mathbb{R}^{n\times d}$), the attended feature $f \in \mathbb{R}^{1\times d}$ is obtained by weighted summation over all values V with respect to the attention learned from q and K:

$$f = A(q, K, V) = \text{softmax}(\frac{qK}{\sqrt{d}})V \tag{1}$$

To further improve the representation capacity of the attended features, *multi-head attention* is introduced in [29], which consists of h paralleled 'heads'. Each head corresponds to an independent scaled dot-product attention function. The attended output features f is given by:

$$f = MA(q, K, V) = [\text{head}_1, \text{head}_2, ..., \text{head}_h]W^o \tag{2}$$

$$\text{head}_j = A(qW_j^Q, KW_j^K, VW_j^V) \tag{3}$$

where $W_j^Q, W_j^K, W_j^V \in \mathbb{R}^{d\times d_h}$ are the projection matrices for the j-th head, and $W_o \in \mathbb{R}^{h*d_h\times d}$. d_h is the dimensionality of the output features from each head. To prevent the multi-head attention model from becoming too large, we usually have $d_h = d/h$. In practice, we can compute the attention function on a set of m queries $Q = [q_1; q_2; ...; q_m] \in \mathbb{R}^{m\times d}$ seamlessly by replacing q with Q in Eq.(2), to obtain the attended output features $F \in \mathbb{R}^{m\times d}$.

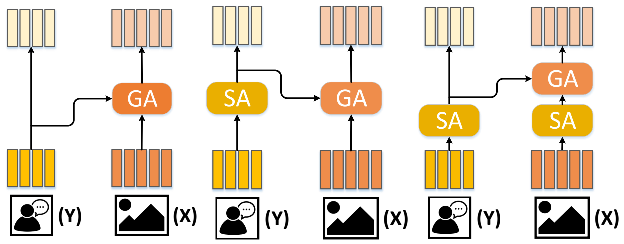

(a) ID(Y)-GA(X,Y) (b) SA(Y)-GA(X,Y) (c) SA(Y)-SGA(X,Y)

Figure 3: Flowcharts of three MCA variants for VQA. (Y) and (X) denote the question and image features respectively.

We build two attention units on top of the multi-head attention to handle the multimodal input features for VQA, namely the *self-attention* (SA) unit and the *guided-attention* (GA) unit. The SA unit (see Figure 2a) is composed of a multi-head attention layer and a pointwise feed-forward layer. Taking one group of input features $X = [x_1; ...; x_m] \in \mathbb{R}^{m\times d_x}$, the multi-head attention learns the pairwise relationship between the paired sample $< x_i, x_j >$ within X and outputs the attended output features $Z \in \mathbb{R}^{m\times d}$ by weighted summation of all the instances in X. The feed-forward layer takes the output features of the multi-head attention layer, and further transforms them through two fully-connected layers with ReLU activation and dropout (FC($4d$)-ReLU-Dropout(0.1)-FC(d)). Moreover, residual connection [12] followed by layer normalization [3] is applied to the outputs of the two layers to facilitate optimization. The GA unit (see Figure 2b) takes two groups of input features $X \in \mathbb{R}^{m\times d_x}$ and $Y = [y_1; ...; y_n] \in \mathbb{R}^{n\times d_y}$, where Y guides the attention learning for X. Note that the shapes of X and Y are flexible, so they can be used to represent the features for different modalities (*e.g.*, questions and images). The GA unit models the pairwise relationship between the each paired sample $< x_i, y_j >$ from X and Y, respectively.

Interpretation: Since the multi-head attention in Eq.(2) plays a key role in the two attention units, we take a closer look at it to see how it works with respect to different types of inputs. For a SA unit with input features X, for each $x_i \in X$, its attended feature $f_i = \text{MA}(x_i, X, X)$ can be understood as *reconstructing* x_i by all the samples in X with respect to their normalized similarities to x_i. Analogously, for a GA unit with input features X and Y, the attended feature $f_i = \text{MA}(x_i, Y, Y)$ for $x_i \in X$ is obtained by reconstructing x_i by all the samples in Y with respect to their normalized cross-modal similarity to x_i.

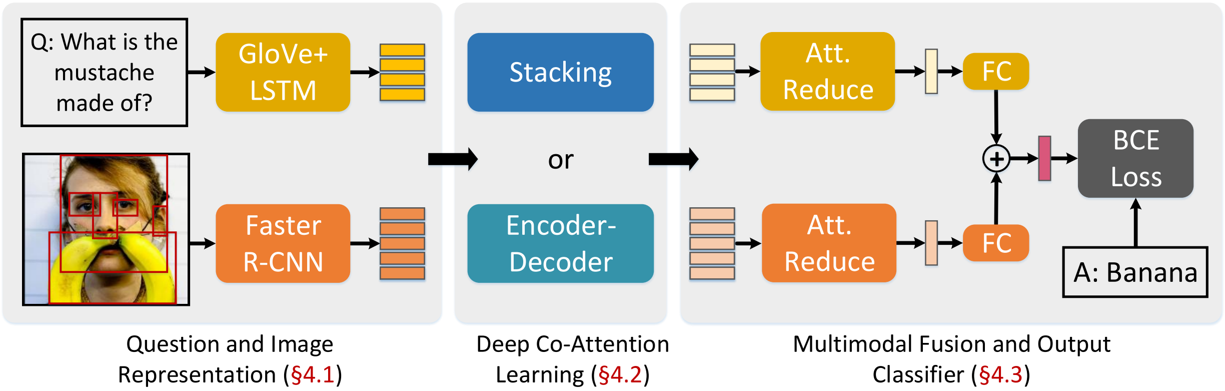

Figure 4: Overall flowchart of the deep Modular Co-Attention Networks (MCAN). In the Deep Co-attention Learning stage, we have two alternative strategies for deep co-attention learning, namely *stacking* and *encoder-decoder*.

3.2. Modular Composition for VQA

Based on the two basic attention units in Figure 2, we composite them to obtain three modular co-attention (MCA) layers (see Figure 3) to handle the multimodal features for VQA. All three MCA layers can be cascaded in depth, such that the outputs of the previous MCA layer can be directly fed to the next MCA layer. This implies that the number of input features is equal to the number of output features without instance reduction.

The ID(Y)-GA(X,Y) layer in Figure 3a is our baseline. In ID(Y)-GA(X,Y), the input question features are directly passed through to the output features with an identity mapping, and the dense inter-modal interaction between each region $x_i \in X$ with each word $y_i \in Y$ is modeled in a GA(X,Y) unit. These interactions are further exploited to obtain the attended image features. Compared to the ID(Y)-GA(X,Y) layer, the SA(Y)-GA(X,Y) layer in Figure 3b adds a SA(Y) unit to model the dense intra-modal interaction between each question word pair $\{y_i, y_j\} \in Y$. The SA(Y)-SGA(X,Y) layer in Figure 3c continues to add a SA(X) unit to the SA(Y)-GA(X,Y) layer to model the intra-modal interaction between each image region pairs $\{x_i, x_j\} \in X$.[1].

Note that the three MCA layers above have not covered all the possible compositions. We have also explored other MCA variants like the symmetric architectures GA(X,Y)-GA(Y,X) and SGA(X,Y)-SGA(Y,X). However, these MCA variants do not report comparative performance, so we do not discuss them further due to space limitations.

4. Modular Co-Attention Networks

In this section, we describe the Modular Co-Attention Networks (MCAN) architecture for VQA. We first explain the image and question feature representation from the input question and image. Then, we propose two deep co-attention models, namely *stacking* and *encoder-decoder*, which consists of multiple MCA layers cascaded in depth to gradually refine the attended image and question features. As we obtained the attended image and question features, we design a simple multimodal fusion model to fuse the multimodal features and finally feed them to a multi-label classifier to predict answer. An overview flowchart of MCAN is shown in Figure 4.

We name the MCAN model with the stacking strategy as MCAN$_{\text{sk}}$-L and the MCAN model with the encoder-decoder strategy as MCAN$_{\text{ed}}$-L, where L is the total number MCA layers cascaded in depth.

4.1. Question and Image Representations

The input image is represented as a set of regional visual features in a bottom-up manner [1]. These features are the intermediate features extracted from a Faster R-CNN model (with ResNet-101 as its backbone) [26] pre-trained on the Visual Genome dataset [18]. We set a confidence threshold to the probabilities of detected objects and obtain a dynamic number of objects $m \in [10, 100]$. For the i-th object, it is represented as a feature $x_i \in \mathbb{R}^{d_x}$ by mean-pooling the convolutional feature from its detected region. Finally, the image is represented as a feature matrix $X \in \mathbb{R}^{m \times d_x}$.

The input question is first tokenized into words, and trimmed to a maximum of 14 words similar to [28, 14]. Each word in the question is further transformed into a vector using the 300-D GloVe word embeddings [25] pre-trained on a large-scale corpus. This results in a sequence of words of size $n \times 300$, where $n \in [1, 14]$ is the number of words in the question. The word embeddings are then passed through a one-layer LSTM network [13] with d_y hidden units. In contrast to [28] which only uses the final state (*i.e.*, the output feature for the last word) as the

[1] In our implementation, we omit the feed-forward layer and norm layer of the SA(X) unit to save memory costs.

question feature, we maintain the output features for all words and output a question feature matrix $Y \in \mathbb{R}^{n \times d_y}$.

To deal with the variable number of objects m and variable question length n, we use zero-padding to fill X and Y to their maximum sizes (*i.e.*, $m = 100$ and $n = 14$, respectively). During training, we mask the padding logits with $-\infty$ to get zero probability before every softmax layer to avoid the underflow problem.

4.2. Deep Co-Attention Learning

Taking the aforementioned image features X and the question features Y as inputs, we perform deep co-attention learning by passing the input features though a deep co-attention model consisting of L MCA layers cascaded in depth (denoted by $\text{MCA}^{(1)}$, $\text{MCA}^{(2)}$ ... $\text{MCA}^{(L)}$). Denoting the input features for $\text{MCA}^{(l)}$ as $X^{(l-1)}$ and $Y^{(l-1)}$ respectively, their output features are denoted by $X^{(l)}$ and $Y^{(l)}$, which are further fed to the $\text{MCA}^{(l+1)}$ as its inputs in a recursive manner.

$$[X^{(l)}, Y^{(l)}] = \text{MCA}^{(l)}([X^{(l-1)}, Y^{(l-1)}]) \tag{4}$$

For $\text{MCA}^{(1)}$, we set its input features $X^{(0)} = X$ and $Y^{(0)} = Y$, respectively.

Taking the SA(Y)-SGA(X,Y) layer as an example (the other two MCA layers proceed in the same manner), we formulate two deep co-attention models in Figure 5.

The *stacking* model (Figure 5a) simply stacks L MCA layers in depth and outputs $X^{(L)}$ and $Y^{(L)}$ as the final attended image and question features. The *encoder-decoder* model (Figure 5b) is inspired by the Transformer model proposed in [29]. It slightly modifies the stacking model by replacing the input features $Y^{(l)}$ of the GA unit in each $\text{MCA}^{(l)}$ with the question features $Y^{(L)}$ from the last MCA layer. The encoder-decoder strategy can be understood as an encoder to learn the attended question features $Y^{(L)}$ with L stacked SA units and a decoder to use $Y^{(L)}$ to learn the attended image features $X^{(L)}$ with stacked SGA units.

The two deep models are of the same size with the same L. As a special case that $L = 1$, the two models are strictly equivalent to each other.

4.3. Multimodal Fusion and Output Classifier

After the deep co-attention learning stage, the output image features $X^{(L)} = [x_1^{(L)}; ...; x_m^{(L)}] \in \mathbb{R}^{m \times d}$ and question features $Y^{(L)} = [y_1^{(L)}; ...; y_n^{(L)}] \in \mathbb{R}^{n \times d}$ already contain rich information about the attention weights over the question words and image regions. Therefore, we design an attentional reduction model with a two-layer MLP (FC(d)-ReLU-Dropout(0.1)-FC(1)) for $Y^{(L)}$ (or $X^{(L)}$) to obtain its attended feature $\tilde{y}$ (or $\tilde{x}$). Taking $X^{(L)}$ as an

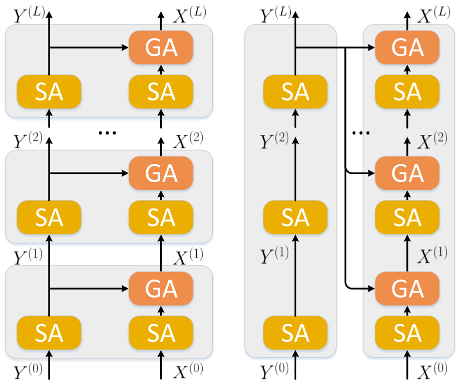

(a) Stacking (b) Encoder-Decoder

Figure 5: Two deep co-attention models based on a cascade of MCA layers (*e.g.*, SA(Y)-SGA(X,Y)).

example, the attended feature $\tilde{x}$ is obtained as follows:

$$\alpha = \text{softmax}(\text{MLP}(X^{(L)}))$$
$$\tilde{x} = \sum_{i=1}^{m} \alpha_i x_i^{(L)} \tag{5}$$

where $\alpha = [\alpha_1, \alpha_2, ..., \alpha_m] \in \mathbb{R}^m$ are the learned attention weights. We can obtain the attended feature $\tilde{y}$ for $Y^{(L)}$ using an independent attentional reduction model by analogy.

Using the computed $\tilde{y}$ and $\tilde{x}$, we design the linear multimodal fusion function as follows:

$$z = \text{LayerNorm}(W_x^T \tilde{x} + W_y^T \tilde{y}) \tag{6}$$

where $W_x, W_y \in \mathbb{R}^{d \times d_z}$ are two linear projection matrices. d_z is the common dimensionality of the fused feature. LayerNorm is used here to stabilize training [3].

The fused feature z is projected into a vector $s \in \mathbb{R}^N$ followed by a sigmoid function, where N is the number of the most frequent answers in the training set. Following [28], we use binary cross-entropy (BCE) as the loss function to train an N-way classifier on top of the fused feature z.

5. Experiments

In this section, we conduct experiments to evaluate the performance of our MCAN models on the largest VQA benchmark dataset, VQA-v2 [11]. Since the different MCA variants and deep co-attention models may influence final performance, we perform extensive quantitative and qualitative ablation studies to explore the reasons why MCAN performs well. Finally, with the optimal hyper-parameters, we compare our best model with current state-of-the-art models under the same settings.

5.1. Datasets

VQA-v2 is the most commonly used VQA benchmark dataset [11]. It contains human-annotated question-answer

(a) **MCA Variants:** Accuracies of the MCAN model with different MCA variants under **one** layer. ID(Y)-GA(X,Y), SA(Y)-GA(X,Y) and SA(Y)-SGA(X,Y) denote the three MCA variants w/ or w/o the SA units for image and question (see Figure 3). Since the stacking and the encoder-decoder strategies are equivalent under one layer, we do not distinguish them.

Model	All	Y/N	Num	Other
ID(Y)-GA(X,Y)	64.8	82.5	44.7	56.7
SA(Y)-GA(X,Y)	65.2	82.9	44.8	57.1
SA(Y)-SGA(X,Y)	**65.4**	**83.2**	**44.9**	**57.2**

(b) **Stacking *vs.* Encoder-decoder:** Overall accuracies and model sizes (*i.e.*, number of parameters) of the MCAN$_{sk}$-L models and the MCAN$_{ed}$-L models, where number of layers $L \in \{2, 4, 6, 8\}$. With the same L, the sizes of the two models are equal.

L	MCAN$_{sk}$	MCAN$_{ed}$	Size
2	66.1	66.2	27M
4	66.7	66.9	41M
6	66.8	**67.2**	56M
8	66.8	**67.2**	68M

(c) **Question Representations:** Accuracies of the MCAN$_{ed}$-6 model with different question representations. Rand$_{ft}$ means the word embeddings are initialized randomly and then fine-tuned. PE denotes the positional encoding [29]. GloVe$_{pt+ft}$ and GloVe$_{pt}$ mean the word embeddings are pre-trained with GloVe, while GloVe$_{pt+ft}$ is additionally fine-tuned.

Model	All	Y/N	Num	Other
Rand$_{ft}$ + PE	65.6	83.0	47.9	57.1
GloVe$_{pt}$ + PE	67.0	84.6	**49.4**	58.2
GloVe$_{pt}$ + LSTM	67.1	**84.8**	**49.4**	58.4
GloVe$_{pt+ft}$ + LSTM	**67.2**	**84.8**	49.3	**58.6**

Table 1: Ablation experiments for MCAN. All the reported results are evaluated on the *val* split.

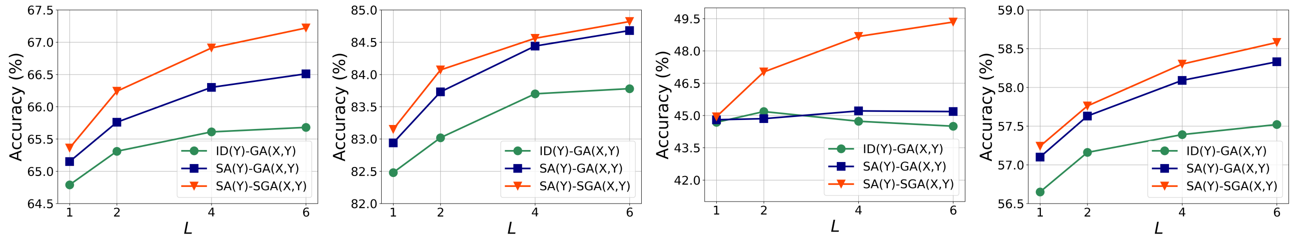

(a) All (b) Y/N (c) Num (d) Other

Figure 6: The overall and per-type accuracies of the MCAN$_{ed}$-L models equipped with different MCA variants, where the number of layers $L \in \{1, 2, 4, 6\}$. All the reported results are evaluated on the *val* split.

pairs relating to the images from the MS-COCO dataset [19], with 3 questions per image and 10 answers per question. The dataset is split into three: *train* (80k images and 444k QA pairs); *val* (40k images and 214k QA pairs); and *test* (80k images and 448k QA pairs). Additionally, there are two test subsets called *test-dev* and *test-standard* to evaluate model performance online. The results consist of three per-type accuracies (*Yes/No*, *Number*, and *Other*) and an overall accuracy.

5.2. Implementation Details

The hyper-parameters of our model used in the experiments are as follows. The dimensionality of input image features d_x, input question features d_y, and fused multi-modal features d_z are 2,048, 512, and 1,024, respectively. Following the suggestions in [29], the latent dimensionality d in the multi-head attention is 512, the number of heads h is set to 8, and the latent dimensionality for each head is $d_h = d/h = 64$. The size of the answer vocabulary is set to $N = 3,129$ using the strategy in [28]. The number of MCA layers is $L \in \{1, 2, 4, 6, 8\}$.

To train the MCAN model, we use the Adam solver [17] with $\beta_1 = 0.9$ and $\beta_2 = 0.98$. The base learning rate is set to $\min(2.5te^{-5}, 1e^{-4})$, where t is the current epoch number starting from 1. After 10 epochs, the learning rate is decayed by 1/5 every 2 epochs. All the models are trained up to 13 epochs with the same batch size 64. For the results on the *val* split, only the *train* split is used for training. For the results on the *test-dev* or *test-standard* splits, both *train* and *val* splits are used for training, and a subset of VQA samples from Visual Genome [18] is also used as the augmented dataset to facilitate training.

5.3. Ablation Studies

We run a number of ablations to investigate the reasons why MCAN is effective. The results shown in Table 1 and Figure 6 are discussed in detail below.

MCA Variants: From the results in Table 1a, we can see that SA(Y)-GA(X,Y) outperforms ID(Y)-GA(X,Y) for all answer types. This verifies that modeling self-attention for question features benefits VQA performance, which is consistent with previous works [33]. Moreover, we can see that SA(Y)-SGA(X,Y) also outperforms SA(Y)-GA(X,Y). This reveals, for the first time, that modeling self-attention for image features is meaningful. Therefore, we use SA(Y)-SGA(X,Y) as our default MCA in the following experiments unless otherwise stated.

Stacking *vs.* Encoder-Decoder: From the results in Table 1b, we can see that with increasing L, the performances of both deep co-attention models steadily improve and finally saturate at $L = 6$. The saturation can be explained by the unstable gradients during training when $L > 6$, which

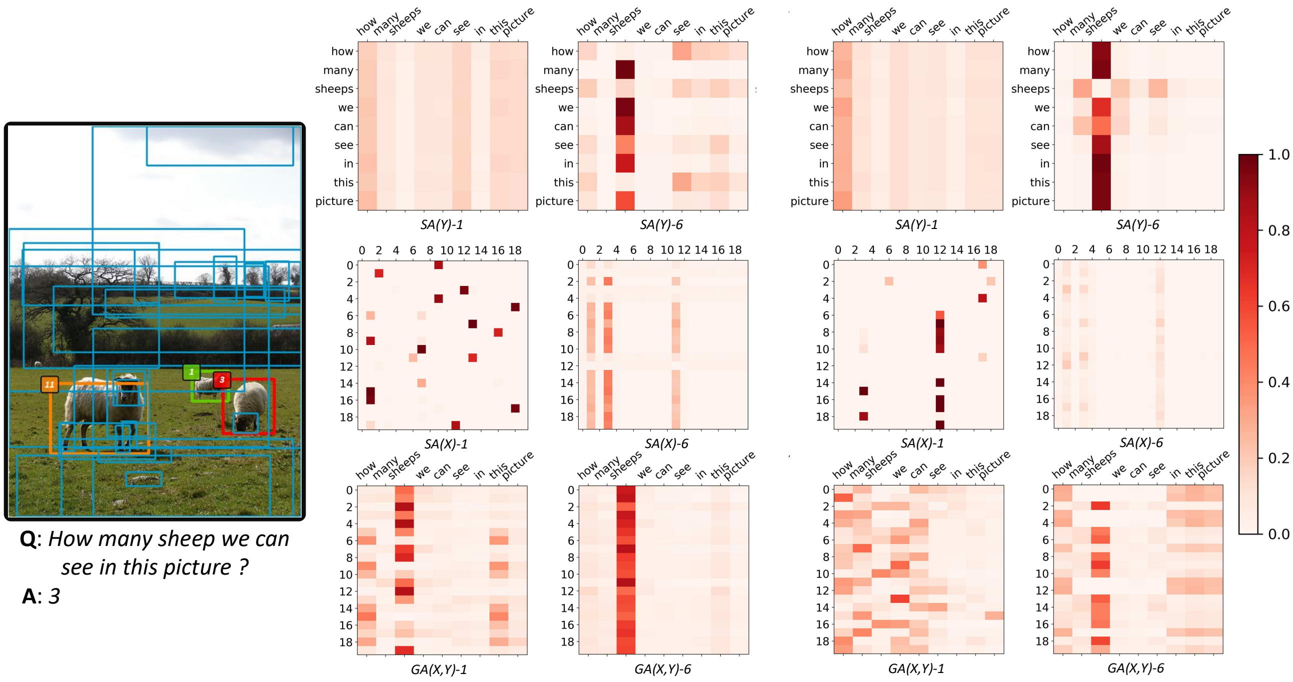

(a) Encoder-Decoder (**P**: 3) (b) Stacking (**P**: 3)

Figure 7: Visualizations of the learned attention maps ($\mathrm{softmax}(qK/\sqrt{d})$) of the attention units from typical layers. SA(Y)-l, SA(X)-l and GA(X,Y)-l denote the question self-attention, image self-attention, and question guided-attention from the l-th layer, respectively. Q, A, P denote the question, answer and prediction respectively. The index within [0-19] shown on the axes of the attention maps corresponds to each object in the image (20 objects in total) . For better visualization effect, we highlight three objects in the image that are related to the answer (*i.e.*, sheep).

makes the optimization difficult. Similar observations are also reported by [5]. Furthermore, the encoder-decoder model steadily outperforms the stacking model, especially when L is large. This is because the learned self-attention from an early SA(Y) unit is inaccurate compared to that from the last SA(Y) unit. Directly feeding it to a GA(X,Y) unit may damage the learned guided-attention for images. The visualization in §5.4 supports this explanation. Finally, MCAN is much more parametric-efficient than other approaches, with MCAN_{ed}-2 (27M) reporting a 66.2% accuracy, BAN-4 (45M) a 65.8% accuracy [14], and MFH (116M) a 65.7% accuracy [33]. More in-depth comparisons can be found in the supplementary material.

MCA *vs.* Depth: In Figure 6, we show the detailed performance of MCAN_{ed}-L with different MCA variants. With increasing L, the performance gaps between the three variants increases. Furthermore, an interesting phenomenon occurs in Figure 6c. When $L = 6$, the *number* type accuracy of the ID(Y)-GA(X,Y) and SA(Y)-GA(X,Y) models are nearly identical, while the SA(Y)-SGA(X,Y) model reports a 4.5-point improvement over them. This verifies that self-attention for images plays a key role in object counting.

Question Representations: Table 1c summarizes ablation experiments on different question representations. We can see that using the word embeddings pre-trained by GloVe [25] significantly outperforms that by random initialization. Other trick like fine-tuning the GloVe embeddings or replacing the position encoding [29] with a LSTM network to model the temporal information can slightly improve the performance further.

5.4. Qualitative Analysis

In Figure 7, we visualize the learned attentions from MCAN_{sk}-6 and MCAN_{ed}-6. Due to space limitations, we only show one example and visualize six attention maps from different attention units and different layers. More visualizations can be found in the supplementary material. From the results, we have the following observations.

Question Self-Attention SA(Y): The attention maps of SA(Y)-1 form vertical stripes, and the words like 'how' and 'see' obtain large attention weights. This unit acts as a question type classifier. Besides, the large values in the attention maps of SA(Y)-6 occur in the column 'sheep'. This reveals that all the attended features tend to use the feature of 'sheep' for reconstruction. That is to say, the keyword 'sheep' is identified correctly.

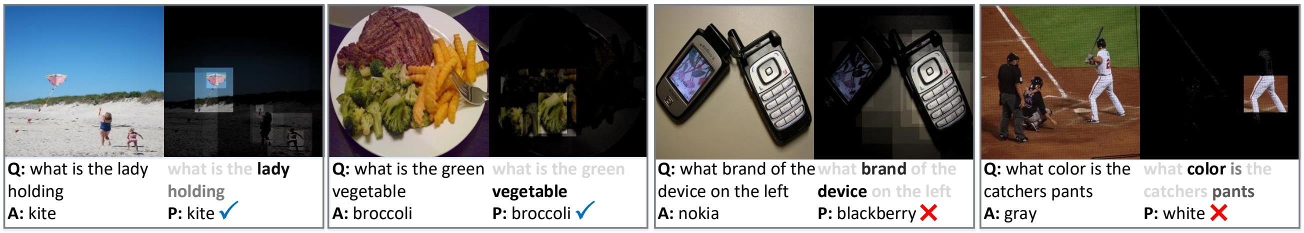

Figure 8: Typical examples of the learned image and question attentions by Eq.(5). For each example, the image, question (Q) and answer (A) are presented on the left; the learned image attention, question attention and prediction (P) are presented next to them. The brightness of regions and darkness of words represent their importance in the attention weights.

Image Self-Attention SA(X): Values in the attention maps of SA(X)-1 are uniformly distributed, suggesting that the key objects for sheep are unclear. The large values in the attention maps of SA(X)-6 occur on the 1st, 3rd, and 11th columns, which correspond to the three sheep in the image. This explains why introducing SA(X) can greatly improve object counting performance.

Question Guided-Attention GA(X,Y): The attention maps of GA(X,Y)-1 do not focus on the current objects in the image; and the attention maps of GA(X,Y)-6 tend to focus on all values in the 'sheep' column. This can be explained by the fact that the input features have been reconstructed by the sheep features in SA(X)-6. Moreover, the GA(X,Y) units of the stacking model contain much more noise than the encoder-decoder model. This verifies our hypothesis presented in §5.3.

In Figure 8, we also visualize the final image and question attentions learned by Eq.(5). For the correctly predicted examples, the learned question and image attentions are usually closely focus on the key words and the most relevant image regions (*e.g.*, the word 'holding' and the region of 'hand' in the first example, and the word 'vegetable' and the region of 'broccoli' in the second example). From the incorrect examples, we can draw some weaknesses of our approach. For example, it occasionally makes mistakes in distinguishing the key words in questions (*e.g.*, the word 'left' in the third example and the word 'catcher' in the last example). These observations are useful to guide further improvements in the future.

5.5. Comparison with State-of-the-Art

By taking the ablation results into account, we compare our best single model MCAN_{ed}-6 with the current state-of-the-art methods in Table 2. Using the same bottom-up attention visual features [1], MCAN_{ed}-6 significantly outperforms the current best approach BAN [14] by 1.1 points in terms of overall accuracy. Compared to BAN+Counter [14], which additionally introduces the counting module [35] to significantly improve object counting performance, our model is still 0.6 points higher. Moreover, our method obtains comparable object counting performance (*i.e.*, the number type) to BAN+Counter, and in doing so does not use any auxiliary information like the bounding-box coordinates of each object [35]. This suggests that MCAN is more general that can naturally learn to *deduplicate* the redundant objects based on the visual features alone. The comparative results with model ensembling are demonstrated in the supplementary material.

Table 2: Accuracies of **single-model** on the *test-dev* and *test-standard* splits to compare with the state-of-the-art methods. All the methods use the same bottom-up attention visual features [1], and are trained on the *train+val+vg* sets (*vg* denotes the augmented VQA samples from Visual Genome). The best results on both splits are bolded.

Model	Test-dev				Test-std
	All	Y/N	Num	Other	All
Bottom-Up [28]	65.32	81.82	44.21	56.05	65.67
MFH [33]	68.76	84.27	49.56	59.89	-
BAN [14]	69.52	85.31	50.93	60.26	-
BAN+Counter [14]	70.04	85.42	**54.04**	60.52	70.35
MCAN_{ed}-6	**70.63**	**86.82**	53.26	**60.72**	**70.90**

6. Conclusions

In this paper, we present a novel deep Modular Co-Attention Network (MCAN) for VQA. MCAN consists of a cascade of modular co-attention (MCA) layers, each of which consists of the self-attention and guided-attention units to model the intra- and inter-modal interactions synergistically. By stacking MCA layers in depth using the encoder-decoder strategy, we obtain a deep MCAN model that achieves new state-of-the-art performance for VQA.

Acknowledgments

This work was supported in part by National Natural Science Foundation of China under Grant 61702143, Grant 61836002, Grant 61622205, and in part by the Australian Research Council Projects under Grant FL-170100117, Grant DP-180103424 and Grant IH-180100002.

References

[1] Peter Anderson, Xiaodong He, Chris Buehler, Damien Teney, Mark Johnson, Stephen Gould, and Lei Zhang. Bottom-up and top-down attention for image captioning and visual question answering. *IEEE Conference on Computer Vision and Pattern Recognition (CVPR)*, pages 6077–6086, 2018. 1, 2, 4, 8

[2] Stanislaw Antol, Aishwarya Agrawal, Jiasen Lu, Margaret Mitchell, Dhruv Batra, C Lawrence Zitnick, and Devi Parikh. Vqa: Visual question answering. In *International Conference on Computer Vision (ICCV)*, pages 2425–2433, 2015. 1, 2

[3] Jimmy Lei Ba, Jamie Ryan Kiros, and Geoffrey E Hinton. Layer normalization. *arXiv preprint arXiv:1607.06450*, 2016. 3, 5

[4] Dzmitry Bahdanau, Kyunghyun Cho, and Yoshua Bengio. Neural machine translation by jointly learning to align and translate. *arXiv preprint arXiv:1409.0473*, 2014. 1

[5] Ankur Bapna, Mia Xu Chen, Orhan Firat, Yuan Cao, and Yonghui Wu. Training deeper neural machine translation models with transparent attention. *arXiv preprint arXiv:1808.07561*, 2018. 7

[6] Hedi Ben-Younes, Rémi Cadene, Matthieu Cord, and Nicolas Thome. Mutan: Multimodal tucker fusion for visual question answering. In *International Conference on Computer Vision (ICCV)*, pages 2612–2620, 2017. 2

[7] Kan Chen, Jiang Wang, Liang-Chieh Chen, Haoyuan Gao, Wei Xu, and Ram Nevatia. Abc-cnn: An attention based convolutional neural network for visual question answering. *arXiv preprint arXiv:1511.05960*, 2015. 1, 2

[8] Jan K Chorowski, Dzmitry Bahdanau, Dmitriy Serdyuk, Kyunghyun Cho, and Yoshua Bengio. Attention-based models for speech recognition. In *Advances in neural information processing systems (NIPS)*, pages 577–585, 2015. 1

[9] Jeffrey Donahue, Lisa Anne Hendricks, Sergio Guadarrama, Marcus Rohrbach, Subhashini Venugopalan, Kate Saenko, and Trevor Darrell. Long-term recurrent convolutional networks for visual recognition and description. In *IEEE Conference on Computer Vision and Pattern Recognition (CVPR)*, pages 2625–2634, 2015. 1

[10] Akira Fukui, Dong Huk Park, Daylen Yang, Anna Rohrbach, Trevor Darrell, and Marcus Rohrbach. Multimodal compact bilinear pooling for visual question answering and visual grounding. *arXiv preprint arXiv:1606.01847*, 2016. 1, 2

[11] Yash Goyal, Tejas Khot, Douglas Summers-Stay, Dhruv Batra, and Devi Parikh. Making the v in vqa matter: Elevating the role of image understanding in visual question answering. *IEEE Conference on Computer Vision and Pattern Recognition (CVPR)*, pages 6904–6913, 2017. 2, 5

[12] Kaiming He, Xiangyu Zhang, Shaoqing Ren, and Jian Sun. Deep residual learning for image recognition. *IEEE Conference on Computer Vision and Pattern Recognition (CVPR)*, pages 770–778, 2016. 3

[13] Sepp Hochreiter and Jürgen Schmidhuber. Long short-term memory. *Neural computation*, 9(8):1735–1780, 1997. 4

[14] Jin-Hwa Kim, Jaehyun Jun, and Byoung-Tak Zhang. Bilinear attention networks. *Advances in neural information processing systems (NIPS)*, 2018. 1, 2, 4, 7, 8

[15] Jin-Hwa Kim, Sang-Woo Lee, Donghyun Kwak, Min-Oh Heo, Jeonghee Kim, Jung-Woo Ha, and Byoung-Tak Zhang. Multimodal residual learning for visual qa. In *Advances in neural information processing systems (NIPS)*, pages 361–369, 2016. 2

[16] Jin-Hwa Kim, Kyoung Woon On, Woosang Lim, Jeonghee Kim, Jung-Woo Ha, and Byoung-Tak Zhang. Hadamard Product for Low-rank Bilinear Pooling. In *International Conference on Learning Representation (ICLR)*, 2017. 1, 2

[17] Diederik P Kingma and Jimmy Ba. Adam: A method for stochastic optimization. *arXiv preprint arXiv:1412.6980*, 2014. 6

[18] Ranjay Krishna, Yuke Zhu, Oliver Groth, Justin Johnson, Kenji Hata, Joshua Kravitz, Stephanie Chen, Yannis Kalantidis, Li-Jia Li, David A Shamma, et al. Visual genome: Connecting language and vision using crowdsourced dense image annotations. *arXiv preprint arXiv:1602.07332*, 2016. 4, 6

[19] Tsung-Yi Lin, Michael Maire, Serge Belongie, James Hays, Pietro Perona, Deva Ramanan, Piotr Dollár, and C Lawrence Zitnick. Microsoft coco: Common objects in context. In *European Conference on Computer Vision (ECCV)*, pages 740–755, 2014. 6

[20] Jiasen Lu, Jianwei Yang, Dhruv Batra, and Devi Parikh. Hierarchical question-image co-attention for visual question answering. In *Advances in neural information processing systems (NIPS)*, pages 289–297, 2016. 1, 2

[21] Mateusz Malinowski and Mario Fritz. A multi-world approach to question answering about real-world scenes based on uncertain input. In *Advances in neural information processing systems (NIPS)*, pages 1682–1690, 2014. 1

[22] Volodymyr Mnih, Nicolas Heess, Alex Graves, et al. Recurrent models of visual attention. In *Advances in neural information processing systems (NIPS)*, pages 2204–2212, 2014. 1

[23] Hyeonseob Nam, Jung-Woo Ha, and Jeonghee Kim. Dual attention networks for multimodal reasoning and matching. *arXiv preprint arXiv:1611.00471*, 2016. 1, 2

[24] Duy-Kien Nguyen and Takayuki Okatani. Improved fusion of visual and language representations by dense symmetric co-attention for visual question answering. *IEEE Conference on Computer Vision and Pattern Recognition (CVPR)*, pages 6087–6096, 2018. 1, 2

[25] Jeffrey Pennington, Richard Socher, and Christopher D Manning. Glove: Global vectors for word representation. In *EMNLP*, pages 1532–1543, 2014. 4, 7

[26] Shaoqing Ren, Kaiming He, Ross Girshick, and Jian Sun. Faster r-cnn: Towards real-time object detection with region proposal networks. In *Advances in neural information processing systems (NIPS)*, pages 91–99, 2015. 4

[27] Kevin J Shih, Saurabh Singh, and Derek Hoiem. Where to look: Focus regions for visual question answering. In *IEEE Conference on Computer Vision and Pattern Recognition (CVPR)*, pages 4613–4621, 2016. 1

[28] Damien Teney, Peter Anderson, Xiaodong He, and Anton van den Hengel. Tips and tricks for visual question answering: Learnings from the 2017 challenge. *arXiv preprint arXiv:1708.02711*, 2017. 4, 5, 6, 8

[29] Ashish Vaswani, Noam Shazeer, Niki Parmar, Jakob Uszkoreit, Llion Jones, Aidan N Gomez, Łukasz Kaiser, and Illia Polosukhin. Attention is all you need. In *Advances in Neural Information Processing Systems*, pages 6000–6010, 2017. 2, 3, 5, 6, 7

[30] Kelvin Xu, Jimmy Ba, Ryan Kiros, Kyunghyun Cho, Aaron C Courville, Ruslan Salakhutdinov, Richard S Zemel, and Yoshua Bengio. Show, attend and tell: Neural image caption generation with visual attention. In *International Conference on Machine Learning (ICML)*, volume 14, pages 77–81, 2015. 1

[31] Zichao Yang, Xiaodong He, Jianfeng Gao, Li Deng, and Alex Smola. Stacked attention networks for image question answering. In *IEEE Conference on Computer Vision and Pattern Recognition (CVPR)*, pages 21–29, 2016. 2

[32] Zhou Yu, Jun Yu, Jianping Fan, and Dacheng Tao. Multi-modal factorized bilinear pooling with co-attention learning for visual question answering. *IEEE International Conference on Computer Vision (ICCV)*, pages 1839–1848, 2017. 2

[33] Zhou Yu, Jun Yu, Chenchao Xiang, Jianping Fan, and Dacheng Tao. Beyond bilinear: Generalized multimodal factorized high-order pooling for visual question answering. *IEEE Transactions on Neural Networks and Learning Systems*, 29(12):5947–5959, 2018. 1, 2, 6, 7, 8

[34] Zhou Yu, Jun Yu, Chenchao Xiang, Zhou Zhao, Qi Tian, and Dacheng Tao. Rethinking diversified and discriminative proposal generation for visual grounding. *International Joint Conference on Artificial Intelligence (IJCAI)*, pages 1114–1120, 2018. 1

[35] Yan Zhang, Jonathon Hare, and Adam Prügel-Bennett. Learning to count objects in natural images for visual question answering. *International Conference on Learning Representation (ICLR)*, 2018. 8

[36] Zhou Zhao, Zhu Zhang, Shuwen Xiao, Zhou Yu, Jun Yu, Deng Cai, Fei Wu, and Yueting Zhuang. Open-ended long-form video question answering via adaptive hierarchical reinforced networks. In *International Joint Conference on Artificial Intelligence (IJCAI)*, pages 3683–3689, 2018. 1

[37] Bolei Zhou, Yuandong Tian, Sainbayar Sukhbaatar, Arthur Szlam, and Rob Fergus. Simple baseline for visual question answering. *arXiv preprint arXiv:1512.02167*, 2015. 2
