# Supplementary material for: Joint embedding VQA model based on dynamic word vector
Source: PeerJ Comput Sci. 2021 Mar 3;7:e353. doi: 10.7717/peerj-cs.353 (PMC7959642; doi:10.7717/peerj-cs.353)
Supplement: Supplemental Information 1 [file peerj-cs-07-353-s001.zip › Explicit knowledge-based reasoning for visual question answering.pdf]

arXiv:1511.02570v2 [cs.CV] 12 Nov 2015

Explicit Knowledge-based Reasoning for Visual Question Answering

Peng Wang*, Qi Wu*, Chunhua Shen, Anton van den Hengel, Anthony Dick
School of Computer Science, The University of Adelaide

November 13, 2015

Abstract

We describe a method for visual question answering which is capable of reasoning about contents of an image on the basis of information extracted from a large-scale knowledge base. The method not only answers natural language questions using concepts not contained in the image, but can provide an explanation of the reasoning by which it developed its answer. The method is capable of answering far more complex questions than the predominant long short-term memory-based approach, and outperforms it significantly in the testing. We also provide a dataset and a protocol by which to evaluate such methods, thus addressing one of the key issues in general visual question answering.

1 Introduction

Visual Question Answering (VQA) requires that a method be able to interactively answer questions about images. The questions are typically posed in natural language, as are the answers. The problem requires image understanding, natural language processing, and a means by which to relate images and text. More importantly, however, the interactivity implied by the problem means that it cannot be determined beforehand which questions will be asked. This requirement to answer a wide range of image-based questions, on the fly, means that the problem is closely related to some of the ongoing challenges in Artificial Intelligence [18].

*The first two authors contributed to this work equally.

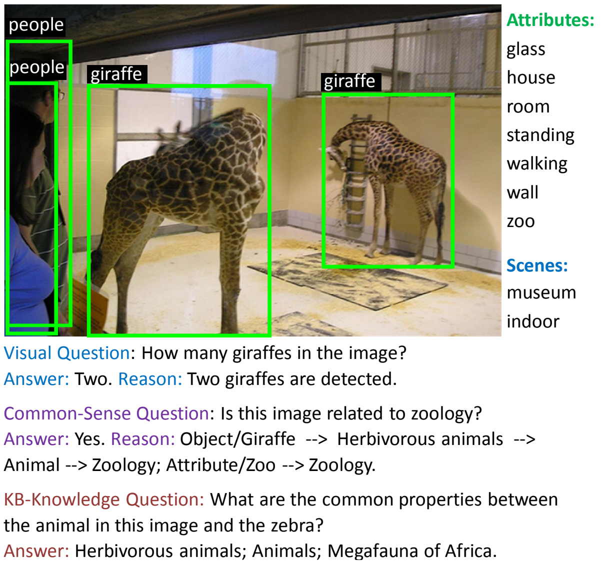

Figure 1: A real example of the proposed KB-VQA dataset and the results given by Ahab, the proposed VQA approach. The questions in the collected dataset are separated into three classes with different knowledge levels: "Visual", "Common-sense" and "KB-knowledge". Our approach answers questions by extracting several types of visual concepts (object, attribute and scene class) from an image and aligning them to large-scale structured knowledge bases. Apart from answers, our approach can also provide reasons/explanations for certain types of questions.

Despite the implied need to perform general reasoning about the content of images, most VQA methods perform no explicit reasoning at all (see, for example, [1, 17, 31, 33]). The predominant method [17, 31] is based on forming a direct connection between a convolutional neural network (CNN) [24, 36, 37] to perform the image analysis, and a Long Short-Term Memory (LSTM) [21] network to process the text.

This approach performs very well in answering simple questions directly related to the content of the image, such as 'What color is the ...?', or 'How many ... are there?'.

There are a number of problems with the LSTM approach, however. The first is that the method does not explain how it arrived at its answer. This means that it is impossible to tell whether it is answering the question based on image information, or just the prevalence of a particular answer in the training set. The second problem is that the amount of prior information that can be encoded within a LSTM system is very limited. DBpedia [2], with millions of concepts and hundred millions of relationships, contains a small subset of the information required to truly reason about the world in general. Recording this level of information would require an implausibly large LSTM, and the amount of training data necessary would be completely impractical. The third, and major, problem with the LSTM approach is that it is incapable of explicit reasoning except in very limited situations [34].

We thus propose Ahab[1], a new approach to VQA which is based on explicit reasoning about the content of images. Ahab first detects relevant content in the image, and relates it to information available in a knowledge base. A natural language question is processed into a suitable query which is run over the combined image and knowledge base information. This query may require multiple reasoning steps to satisfy. The response to the query is then processed so as to form the final answer to the question.

This process allows complex questions to be asked which rely on information not available in the image. Examples include questioning the relationships between two images, or asking whether two depicted animals are close taxonomic relatives.

1.1 Background

The first VQA approach [29] proposed to process questions using semantic parsing [26] and obtain answers through Bayesian reasoning. Both [17] and [31] used CNNs to extract image features and relied on LSTMs to encode questions and decode answers. The primary distinction between these approaches is that [17] used independent LSTM networks for question encoding and answer decoding, while [31] used one LSTM for both tasks. Irrespective of the finer details, we label this the LSTM approach.

Datasets are critical to VQA, as the generality of the questions asked, and the amount of training data available, have a large impact on the set of methods applicable. Malinowski and Fritz [30] proposed the DAQUAR dataset, which is mostly composed of questions requiring only visual knowledge. Ren *et al.* [33] constructed a VQA dataset (refer to as TORONTO-QA), which generated questions automatically by transforming captions on MS COCO [27] images. The COCO-VQA dataset [1] is currently the largest VQA dataset, which contains 300K questions and answers about MS COCO images.

Answering general questions posed by humans about images inevitably requires reference to information not contained in the image itself. To an extent this information may be provided by an existing training set such as ImageNet [12], or MS COCO [27] as class labels or image captions. This approach is inflexible and does not scale, however, and cannot provide the wealth of background information required to answer even relatively simple questions about images. This has manifested itself in the fact that it has proven very difficult to generate a set of image-based questions which are simple enough that VQA approaches can actually answer them [1, 30, 33].

Significant advances have been made, however, in the construction of large-scale *structured* Knowledge Bases (KBs) [2, 3, 6, 9, 10, 28, 39]. In structured KBs, knowledge is typically represented by a large number of triples of the form `(arg1,rel,arg2)`, where `arg1` and `arg2` denote two entities in the KB and `rel` denotes a predicate representing the relationship between these two entities. A collection of such triples can be seen as a large interlinked graph. Such triples are often described in terms of a Resource Description Framework [20] (RDF) specification, and housed in a relational database management system (RDBMS), or triple-store, which allows queries over the data. The knowledge that "a cat is

[1]Ahab, the captain in the novel Moby Dick, is either a brilliant visionary, or a deluded fanatic, depending on your perspective.

a domesticated animal", for instance, is stored in an RDF KB by the triple (cat,is-a,domesticated animal). The information in KBs can be accessed efficiently using a query language. In this work we use SPARQL Protocol [32] to query the OpenLink Virtuoso [13] RDBMS. For example, the query ?x:(?x,is-a,domesticated animal) returns all domesticated animals in the graph.

Popular large-scale structured KBs are constructed either by manual-annotation/crowd-sourcing (*e.g.*, DBpedia [2], Freebase [6] and Wikidata [39]), or by automatically extracting from unstructured/semi-structured data (*e.g.*, YAGO [22, 28], OpenIE [3, 14, 15], NELL [9], NEIL [10, 11]). The KB we use here is DBpedia, which contains structured information extracted from Wikipedia. Compared to KBs extracted automatically from unstructured data (such as OpenIE), the data in DBpedia is more accurate and has a well-defined ontology. The method we propose is applicable to any KB that admits SPARQL queries, however, including those listed above and the huge variety of subject-specific RDF databases available.

The advances in structured KBs, have driven an increasing interest in the NLP and AI communities in the problem of natural language question answering using structured KBs (refer to as KB-QA) [4, 7, 8, 16, 23, 25, 41, 26, 38]. The VQA approach which is closest to KB-QA (and to our approach) is that of Zhu *et al.* [43] as they use a KB and RDBMS to answer image-based questions. They build the KB for the purpose, however, using an MRF model, with image features and scene/attribute/affordance labels as nodes. The undirected links between nodes represent mutual compatibility/incompatibility relationships. The KB thus relates specific images to specified image-based quantities to the point where the database schema prohibits recording general information about the world. The queries that this approach can field are crafted in terms of this particular KB, and thus relate to the small number of attributes specified by the schema. The questions are framed in an RDMBS query language, rather than natural language.

1.2 Contribution

Our primary contribution is a method we label Ahab for answering a wide variety of questions about images that require external information to answer. The method accepts questions in natural language, and answers in the same form. It is capable of correctly answering a far broader range of image-based questions than competing methods, and provides an explanation of the reasoning by which it arrived at the answer. Ahab exploits DBpedia as its source of external information, and requires no VQA training data (it does use ImageNet and MS COCO to train the visual concept detector).

We also propose a dataset, and protocol for measuring performance, for general visual question answering. The questions in the dataset are generated by human subjects based on a number of predefined templates. The questions are given one of three labels reflecting the information required to answer them: "Visual", "Common-sense" and "KB-knowledge" (see Fig. 1). Compared to other VQA datasets [1, 27, 30, 33], the questions in the KB-VQA dataset, as a whole, require a higher level of external knowledge to answer. It is expected that humans will require the help of Wikipedia to answer "KB-knowledge" questions. The evaluation protocol requires human evaluation of question answers, as this is the only practical method of testing which does not place undue limits on the questions which can be asked.

2 The KB-VQA Dataset

The KB-VQA dataset has been constructed for the purpose of evaluating the performance of VQA algorithms capable of answering higher knowledge level questions and explicit reasoning about image contents using external information.

2.1 Data Collection

Images We select 700 of the validation images from the MS COCO [27] dataset due to the rich contextual information and diverse object classes therein. The images are selected so as to cover around 150 object

Name	Template	Num.
IsThereAny	Is there any ⟨*concept*⟩?	419
IsImgRelate	Is the image related to ⟨*concept*⟩?	381
WhatIs	What is the ⟨*obj*⟩?	275
ImgScene	What scene does this image describe?	263
ColorOf	What color is the ⟨*obj*⟩?	205
HowMany	How many ⟨*concept*⟩ in this image?	157
ObjAction	What is the ⟨*person/animal*⟩ doing?	147
IsSameThing	Are the ⟨*obj1*⟩ and the ⟨*obj2*⟩ the same thing?	71
MostRelObj	Which ⟨*obj*⟩ is most related to ⟨*concept*⟩?	56
ListObj	List objects found in this image.	54
IsTheA	Is the ⟨*obj*⟩ a ⟨*concept*⟩?	51
SportEquip	List all equipment I might use to play this sport.	48
AnimalClass	What is the ⟨*taxonomy*⟩ of the ⟨*animal*⟩?	46
LocIntro	Where was the ⟨*obj*⟩ invented?	40
YearIntro	When was the ⟨*obj*⟩ introduced?	32
FoodIngredient	List the ingredient of the ⟨food⟩.	31
LargestObj	What is the largest/smallest ⟨*concept*⟩?	27
AreAllThe	Are all the ⟨*obj*⟩ ⟨*concept*⟩?	27
CommProp	List the common properties of the ⟨*obj1*⟩ and ⟨*concept/obj2*⟩.	26
AnimalRelative	List the close relatives of the ⟨*animal*⟩.	17
AnimalSame	Are ⟨*animal1*⟩ and ⟨*animal2*⟩ in the same ⟨*taxonomy*⟩?	17
FirstIntro	Which object was introduced earlier, ⟨*obj1*⟩ or ⟨*concept/obj2*⟩?	8
ListSameYear	List things introduced in the same year as the ⟨*obj*⟩.	4

Table 1: Question templates in descending order of number of instantiations. The total number of questions is 2402. Note that some templates allow questioners to ask the same question in different forms.

classes and 100 scene classes, and typically exhibit 6 to 7 objects each.

Templates Five human subjects (questioners) are asked to generate 3 to 5 question/answer pairs for each of the 700 images, by instantiating the 23 templates shown in Table 1. There are several slots to be filled in these templates:

– ⟨*obj*⟩ is used to specify the visual objects in an image, which can be a single word "object" or its super-class name like "animal", "food" or "vehicle". We also allow questioners to specify a visual object using its size (*e.g.*, small, large) or location (*e.g.*, left, right, top, bottom, center).

– ⟨*person*⟩/⟨*animal*⟩/⟨*food*⟩ is used to specify a person/animal/food with optionally size or location.

– ⟨*concept*⟩ can be filled by any word or phrase which probably corresponds to an entity in DBpedia.

– ⟨*taxonomy*⟩ corresponds to the taxonomy of animals, including kingdom, phylum, class, order, family or genus.

Questions The questions of primary interest here are those require knowledge external to the image to answer. Each question has a label reflecting the human-estimated level of knowledge required to answer it correctly. The "Visual" questions can be answered directly using visual concepts gleaned from ImageNet and MS COCO (such as "Is there a dog in this image?"); "Common-sense" questions should not require an adult to refer to an external source ("How many road vehicles in this image?"); while answering "KB-knowledge" questions is expected to require Wikipedia or similar ("When was the home appliance in this image invented?").

2.2 Data Analysis

Questions From Table 1, we see that the top 5 most frequently used templates are *IsThereAny*, *IsImgRelate*, *WhatIs*, *ImgScene* and *ColorOf*. Some templates

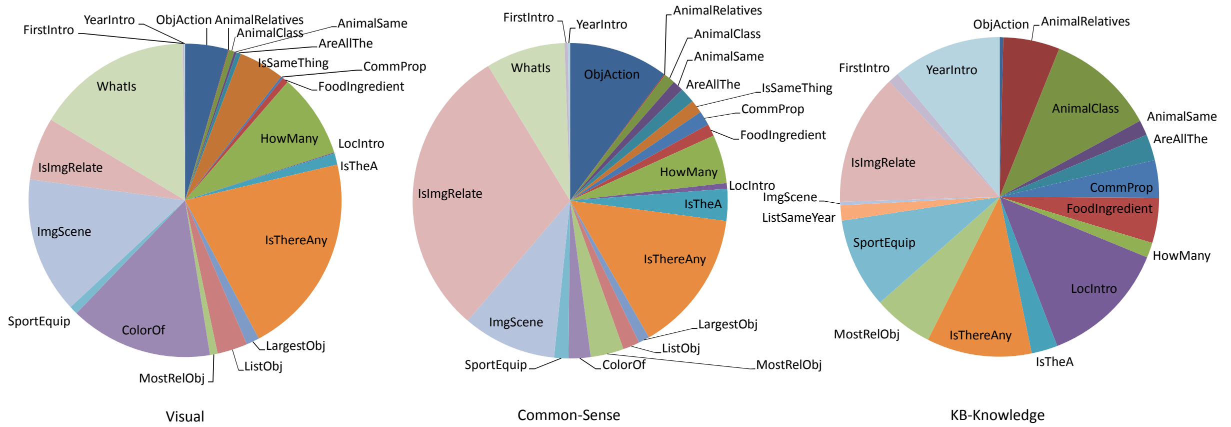

Figure 3: Question template frequencies for different knowledge levels.

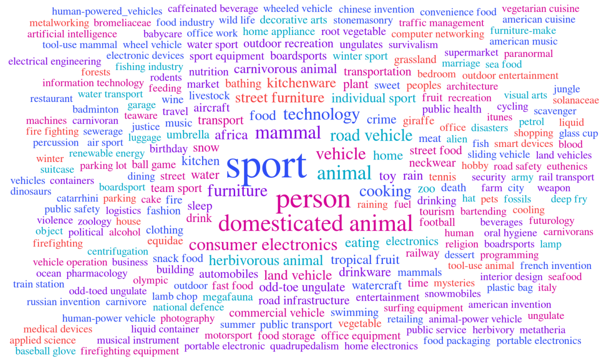

Figure 2: The cloud of 254 ⟨*concept*⟩-phrases for questions in levels "Common-sense" and "KB-knowledge", with size representing the frequency. 55% of these phrases have less than 20 mentions in the 300K questions of the COCO-VQA dataset.

lead to questions that can be answered without any external knowledge, such as *ImgScene* and *ListFound*. But "Common-sense" or "KB-knowledge" is required to analyze the relationship between visual objects and concepts in questions like *IsTheA*, *AreAllThe* and *IsThereAny*. More complex questions like *YearInvent* and *AniamlClass* demand "KB-knowledge". The total number of questions labelled "Visual", "Common-sense" and "KB-knowledge" are 1256, 883 and 263 respectively. So answering around half of the questions will require external knowledge. Fig. 3 shows the distribution of the 23 templates for each question type. Templates *IsImgRelate* and *IsThereAny* cover almost half of the "Common-sense" questions. There are 18 templates shown for "KB-knowledge" questions (as *WhatIs*, *IsSameThing*, *ListObj*, *LargestObj* and *ColorOf* do not appear), which exhibit a more balanced distribution.

In total, 330 different phrases were used by questioners in filling the ⟨*concept*⟩ slot. There were 254 phrases (see Fig. 2) used in questions requiring external knowledge (*i.e.*, "Common-sense" and "KB-knowledge"), 55% of which are mentioned very rarely (less than 20 times) in the 300K questions of the COCO-VQA [1] dataset. For the "KB-knowledge" level, 67 phrases are used and greater than 85% of them have less than 20 mentions in COCO-VQA. Examples of concepts not occurring in COCO-VQA include "logistics", "herbivorous animal", "animal-powered vehicle", "road infrastructure" and "portable electronics".

Compared to other VQA datasets, a large proportion of the questions in KB-VQA require external knowledge to answer. The questions defined in DAQUAR [30] are almost exclusively "Visual" questions, referring to "color", "number" and "physical location of the object". In the TORONTO-QA dataset [33], questions are generated automatically from image captions which describe the major visible content of the image. For the COCO-VQA dataset [1], only 5.5% of questions require adult-level common-sense, and none requires "KB-knowledge" (by observation).

Answers Questions starting with "Is ..." and "Are ..." require logical answers (61% "yes" and 39% "no"). Questions starting with "How many", "What color", "Where" and "When" need to be answered

with number, color, location and time respectively. Of the human answers for “How many” questions, 74% are less than or equal to 5. The most frequent number answers are “1” (58 occurrences), “2” (66) and “3” (44). We also have 16 “How many” questions with human answer “0”. The answers for “What ...” and “List ...” vary significantly, covering a wide range of concepts.

3 The Ahab VQA approach

3.1 RDF Graph Construction

In order to reason about the content of an image we need to amass the relevant information. This is achieved by detecting concepts in the query image and linking them to the relevant parts of the KB.

Visual Concepts Three types of visual concepts are detected in the query image, including:

– *Objects:* We trained two Fast-RCNN [19] detectors on MS COCO 80-class objects [27] and ImageNet 200-class objects [12]. Some classes with low precision were removed from the models, such as “ping-pong ball” and “nail”. The finally merged detector contains 224 object classes, which can be found in the supplementary material.

– *Image Scenes:* The scene classifier is obtained from [42], which is a VGG-16 [36] CNN model trained on the MIT Places205 dataset [42]. In our system, the scene classes corresponding to the top-3 scores are selected.

– *Image Attributes:* In the work of [40], a VGG-16 CNN pre-trained on ImageNet is fine-tuned on the MS COCO image-attribute training data. The vocabulary of attributes defined in [40] covers a variety of high-level concepts related to an image, such as actions, objects, sports and scenes. We select the top-10 attributes for each image.

Linking to the KB Having extracted a set of concepts of interest from the image, we now need to relate them to the appropriate information in the KB. As shown in the left side of Fig. 4, the visual concepts (object, scene and attribute categories) are stored as RDF triples. For example, the information that “The image contains a giraffe

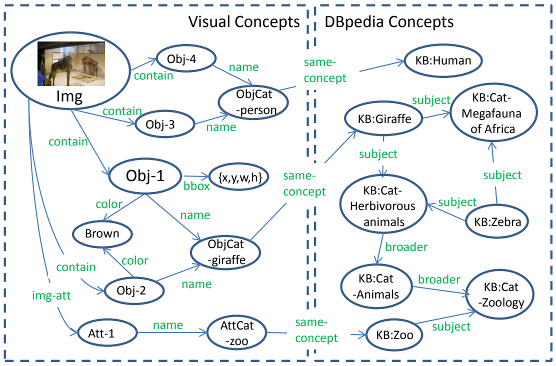

Figure 4: A visualisation of an RDF graph such as might be constructed by Ahab. The illustrated entities are relevant to answering the questions in Fig. 1 (many others not shown for simplicity). Each arrow corresponds to one triple in the graph, with circles representing entities and green text reflecting predicate type. The graph of extracted visual concepts (left side) is linked to DBpedia (right side) by mapping object/attribute/scene categories to DBpedia entities using the predicate `same-concept`.

object” is expressed as: `(Img,contain,Obj-1)` and `(Obj-1,name,ObjCat-giraffe)`. Each visual concept is linked to DBpedia entities with the same semantic meaning (identified through a uniform resource identifier[2] (URI)), for example `(ObjCat-giraffe, same-concept, KB:Giraffe)`. The resulting RDF graph includes all of the relevant information in DBpedia, linked as appropriate to the visual concepts extracted from the query image. This combined image and DBpedia information is then accessed through a local OpenLink Virtuoso [13] RDBMS.

3.2 Answering Questions

Having gathered all of the relevant information from the image and DBpedia, we now use them to answer questions.

Parsing NLQs Given a question posed in natural language, we first need to translate it to a format which can be used to query the RDBMS. Quepy[3] is a Python framework designed within the NLP community to achieve exactly this task. To achieve this

[2] In DBpedia, each entity has a uniform resource identifier (URI). For example, the animal giraffe corresponds to URI: `http://dbpedia.org/resource/Giraffe`.

[3] `http://quepy.readthedocs.org/en/latest/`

Quepy requires a set of templates, framed in terms of regular expressions. It is these templates which form the basis of Table 1. Quepy begins by tagging each word in the question using NLTK [5], which is composed of a tokenizer, a part-of-speech tagger and a lemmatizer. The tagged question is then parsed by a set of regular expressions (regex), each defined for a specific question template. These regular expressions are built using REfO[4] to increase the flexibility of question expression as much as possible. Once a regex matches the question, it will extract the slot-phrases and forward them for further processing. For example, the question in Fig. 5 is matched to template *CommProp* and the slot-phrases for ⟨*obj*⟩ and ⟨*concept*⟩ are "right animal" and "zebra" respectively.

Mapping Slot-Phrases to KB-entities Note that the slot-phrases are still expressed in natural language. The next step is to find the correct correspondences between the slot-phrases and entities in the constructed graph.

− Slots ⟨*obj*⟩/⟨*animal*⟩/⟨*food*⟩ correspond to objects detected in the image, and are identified by comparing provided locations, sizes and names with information in the RDF graph recovered from the image (bounding boxes, for example). This process is heuristic, and forms part of the Quepy rules. In Fig. 5, "right animal" is thus mapped to the entity `Obj-1` in the linked graph (see Fig. 4).

− Phrases in slot ⟨*concept*⟩ need to be mapped to entities in DBpedia. In Ahab this mapping is conducted by string matching between phrases and entity names. The DBpedia predicate `wikiPageRedirects` is used to handle synonyms, capitalizations, punctuation, tenses, abbreviation and misspellings (see the supplementary material for details). In Fig. 5, we can see that the phrase "zebra" is mapped to the KB-entity `KB:Zebra`.

Query Generation With all concepts mapped to KB entities, the next step is to form the appropriate SPARQL queries, depending on the question template. The following several types of DBpedia predicates are used extensively for generating queries and further analysis:

− *Infoboxes* in Wikipedia provide a variety of relationships for different types of entities, such as animal taxonomy and food ingredient.

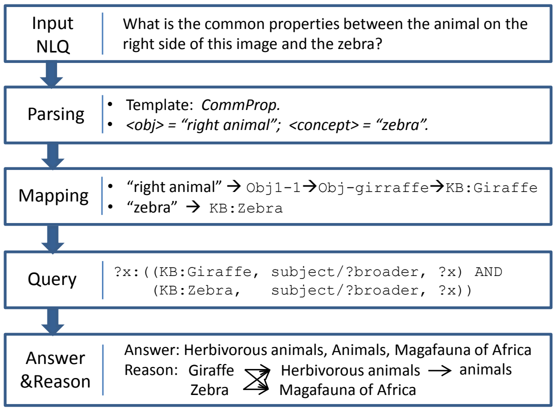

Figure 5: The question processing pipeline. The input question is parsed using a set of NLP tools, and the appropriate template identified. The extracted slot-phrases are then mapped to entities in the KB. Next, KB queries are generated to mine the relevant relationships for the KB-entities. Finally, the answer and reason are generated based on the query results. The predicate `category/?broader` is used to obtain the categories transitively (see [32] for details).

− *Wikilinks* are extracted from the internal links between Wikipedia articles. Compared to Infoboxes, Wikilinks are generic and less precise as the relation property is unknown. There are 162 million such links in the DBpedia 2015-04 dump, which makes the graph somewhat overly-connected. However, we find that counting the number of Wikilinks is still useful for measuring correlation between two entities.

− *Transitive Categories* DBpedia entities are categorized according to the SKOS[5] vocabulary. Non-category entities are linked by predicate `subject` to the category entities they belong to. One category is further linked to its super-category through predicate `broader`. These categories and super-categories (around 1.2 million in the 2015-04 dump) can be found using transitive queries (see [32] and Fig. 5), and are referred to as *transitive categories* here.

To answer the questions *IsThereAny*, *HowMany*, *IsTheA*, *LargestObj* and *AreAllThe*, we need to determine if there is a hyponymy relationship between two entities (*i.e.*, one entity is conceptually a specific

[4] `https://github.com/machinalis/refo`

[5] `http://www.w3.org/2004/02/skos/`

instance of another general entity). This is done by checking if one entity is a transitive category of the other. For question *CommProp*, we collect the transitive categories shared by two entities (see Fig. 5). For questions *IsImgRelate* and *MostRelObj*, the correlation between a visual concept and the concept given in the question is measured based on checking the hyponymy relationship and counting the number of Wikilinks[6]. Answering other templates (*e.g.*, *FoodIngredient*) needs specific types of predicates extracted from Wikipedia infoboxes. The complete list of queries for all templates is given in the supplementary material.

Answer and Reason The last step is to generate answers according to the results of the queries. Post-processing operations can be specified in Python within Quepy, and are needed for some questions, such as *IsImgRelate* and *MostRelObj* (see supplementary material for details).

Note that our system performs searches along the paths from visual concepts to KB concepts. These paths can be used to give "logical reasons" as to how the answer is generated. Especially for questions requiring external knowledge, the predicates and entities on the path give a better understanding of how the relationships are established between visual concepts and KB concepts. Examples of answers and reasons can be seen in Fig. 1 and 5.

4 Experiments

Metrics Performance evaluation in VQA is complicated by the fact that two answers can have no words in common and yet both be perfectly correct. Malinowski and Fritz [29] used the Wu-Palmer similarity (WUPS) to measure the similarity between two words based on their common subsequence in the taxonomy tree. However, this evaluation metric restricts the answer to be a single word. Antol *et al.* [1] provided an evaluation metric for the open-answer task which records the percentage of answers in agreement with ground truth from several human subjects. This evaluation metric requires around 10 ground truth answers for each question, and only partly solves the problem (as indicated by the fact that even human performance is very low in some cases in [1], such as 'Why ...?' questions.).

In our case, the existing evaluation metrics are particularly unsuitable because most of the questions in our dataset are open-ended, especially for the "KB-knowledge" questions. In addition, there is no automated method for assessing the reasons provided by our system. The only alternative is a human-based approach. Hence, we ask 5 human subjects (examiners) to evaluate the results manually. In order to understand the generated answers better, we ask the examiners to give each answer or reason a correctness score as follows: 1: "Totally wrong"; 2: "Slightly wrong"; 3: "Borderline"; 4: "OK"; 5: "Perfect". An answer or reason scored higher than "Borderline" is considered as "right"; otherwise, it is considered as "wrong". We perform this evaluation double-blind, *i.e.* examiners are different from questions/answers providers and do not know the answers source. The protocol for measuring performance is described in the supplementary material.

Evaluation We compare our Ahab system with an approach (which we label LSTM) that encodes both CNN extracted features and questions with an encoder LSTM and generates answers with a decoder LSTM. Specifically, we use the second fully-connected layer (4096-d) of a pre-trained VGG model as the image features, and the LSTM is trained on the training set of COCO-VQA data [1][7]. The LSTM layer contains 256 memory cells in each unit. We also relate "Human" performance for reference.

Table 2 provides the final evaluation results for different question types. Our system outperforms the LSTM on all question types with a final accuracy of 69.6%. Human performance is 92.0%. For question types particularly dependent on KB-knowledge, such as *AnimalClass*, *YearIntro*, *FoodIngredient*, *Comm-*

[6] We count the number of Wikilinks between two entities, but also consider paths through a third entity. See supplementary material for details.

[7] This baseline LSTM achieves 44.93% accuracy on the COCO-VQA validation set, under its evaluation protocol. We note that training LSTM on another dataset provides it an unfair advantage. However, the current KB-VQA dataset is still relatively small and so does not support training large models. The presented dataset will be extended in near future.

Question Type	Accuracy(%)			Correctness (Avg.)		
	LSTM	Ours	Human	LSTM	Ours	Human
IsThereAny	64.9	86.9	93.6	3.6	4.5	4.7
IsImgRelate	57.0	82.2	97.1	3.3	4.2	4.9
WhatIs	26.9	66.9	94.5	2.1	3.7	4.8
ImgScene	30.4	69.6	85.9	2.3	3.8	4.5
ColorOf	14.6	29.8	93.2	1.7	2.5	4.7
HowMany	32.5	56.1	90.4	2.3	3.3	4.6
ObjAction	19.7	57.1	90.5	1.8	3.5	4.7
IsSameThing	54.9	77.5	91.5	3.2	4.2	4.6
MostRelObj	32.1	80.4	92.9	2.3	4.2	4.6
ListObj	1.9	63.0	100	1.1	3.6	4.8
IsTheA	74.5	80.4	92.2	3.9	4.2	4.7
SportEquip	2.1	70.8	79.2	1.2	3.9	4.2
AnimalClass	0.0	87.0	95.7	1.0	4.5	4.8
LocIntro	2.5	67.5	95.0	1.1	3.6	4.8
YearIntro	0.0	46.9	93.8	1.0	2.9	4.8
FoodIngredient	0.0	58.1	74.2	1.0	3.4	4.3
LargestObj	0.0	66.7	96.3	1.0	3.8	4.8
AreAllThe	29.6	63.0	81.5	2.3	3.7	4.3
CommProp	0.0	76.9	76.9	1.0	4.1	4.2
AnimalRelative	0.0	88.2	76.5	1.1	4.4	4.1
AnimalSame	41.2	70.6	94.1	2.6	3.8	4.8
FirstIntro	25.0	25.0	75.0	2.0	1.5	4.1
ListSameYear	25.0	75.0	50.0	1.8	4.2	3.0
Overall	36.2	69.6	92.0	2.5	3.8	4.7

Table 2: Human evaluation results of different methods for different question types. Accuracy is the percentage of correctly answered questions (*i.e.*, correctness scored higher than "Borderline"). The average answer correctness ($\in$ [1,5], the higher the better) for each question type is also listed. We also evaluated human provided answers as a reference.

Prop and *AnimalRelative*, all LSTM-generated answers were marked as "wrong" by examiners. In contrast, our system performs very well on these questions. For example, we achieve 88.2% on questions of type *AnimalRelative*, which is better than human performance. We also outperform humans on the question type *ListSameYear*, which requires knowledge of the year a concept was introduced, and all things introduced in the same year. For the purely "visual" questions such as *WhatIs*, *HowMany* and *ColorOf*, there is still a gap between our proposed system and humans. However, this is mainly caused by the detection error of the object detectors, which is not the focus of this paper. According to the overall average correctness, we achieve 3.8, which lies between "Borderline" and "OK". The LSTM scores only 2.5 while Human achieves 4.7.

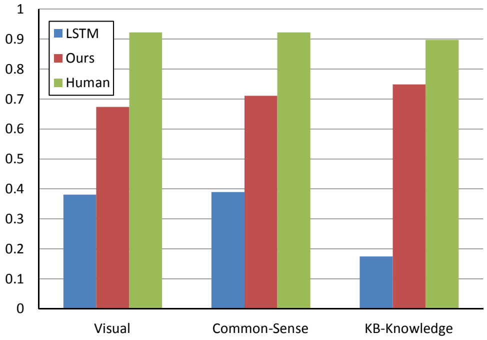

Figure 6: Accuracy of different methods for different knowledge levels. Humans perform almost equally over all three levels. LSTM performs worse for questions requiring higher-level knowledge, whereas Ahab performs better.

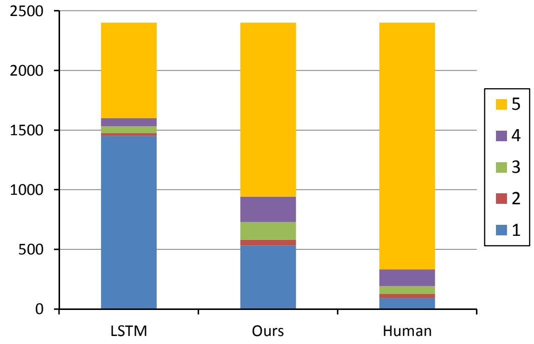

Figure 7: The number of answers that fall into the various correctness levels (1: "Totally wrong"; 2: "Slightly wrong"; 3: "Borderline"; 4: "OK"; 5: "Perfect"), for different methods.

Fig. 6 relates the performance for "Visual", "Common-sense" and "KB-knowledge" questions. The overall trend is same as in Table 2 — Ahab performs better than the LSTM method but not as well as humans. It is not surprising that humans perform almost equally for different knowledge levels, since we allow the human subjects to use Wikipedia to answer the "KB-knowledge" related questions. For the LSTM method, there is a significant decrease in performance as the dependency on external knowledge increases. In contrast, Ahab performs better as the level of external knowledge required increases. *In summary, our system Ahab performs better than LSTM at all three knowledge levels. Furthermore, the performance gap between Ahab and LSTM is more significant for questions requiring external knowledge.*

Fig. 7 indicates the numbers of answers that fall within the different correctness levels. For the LSTM method, more than 50% of the generated answers are

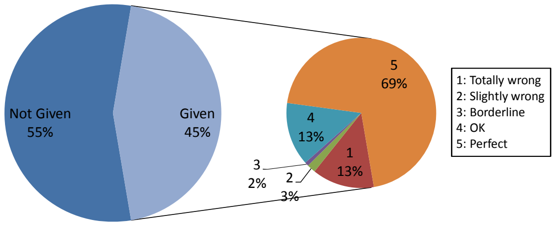

Figure 8: The correctness of the reasons provided by our Ahab system. The left pie chart demonstrates that our system provides reasons for 45% of the questions in the dataset. The right pie shows the distribution of correctness for the given reasons.

grouped to level 1, which is "Totally wrong". For the human performance, only 10% of the answers are not in level 5. Ahab provides the largest portion (around 20%) of answers falling in levels 2, 3 or 4. From the view of the human examiners, the answers given by our Ahab are "softer" than those of other methods. Q1-Q6 of Fig. 9 show some examples of the questions in KB-VQA dataset and the answers and reasons given by Ahab. More examples can be found in the supplementary material.

Reason accuracy Fig. 8 gives an assessment of the quality of the reasons generated by our system. Note that the LSTM cannot provide such information. Since "Visual" questions can be answered by direct interrogation of pixels, we have not coded reasons into the question answering process for the corresponding templates (the reasons would be things like "The corresponding pixels are brown"). Fig. 8 relates the accuracy of the reasons given in the remaining 45% of cases as measured by human examiners using the same protocol. It shows that more than 80% of reasons are marked as being correct (*i.e.*, scored higher than 3). This is significant, as it shows that the method is using valid reasoning to draw its conclusions. Examples of the generated reasons can be found in Fig. 1, Q1-Q3 in Fig. 9 and in the supplementary material.

Extending VQA forms Typically, a VQA problem involves one image and one natural language question (IMG+NLQ). Here we extend VQA to problems involving more images. With this extension, we can ask more interesting questions and more clearly demonstrate the value of using a structured knowledge base. In Fig. 9, we show two types of question involving two images and one natural language question (IMG1+IMG2+NLQ). The first type of question (Q7-Q9) asks for the common properties between two whole images; the second type (Q10-Q12) gives a concept and asks which image is the most related to this concept.

For the first question type, Ahab obtains the answers by searching all common transitive categories shared by the visual concepts extracted from the two query images. For example, although the two images in Q9 are significantly different visually (even at the object level), and share no attributes in common,

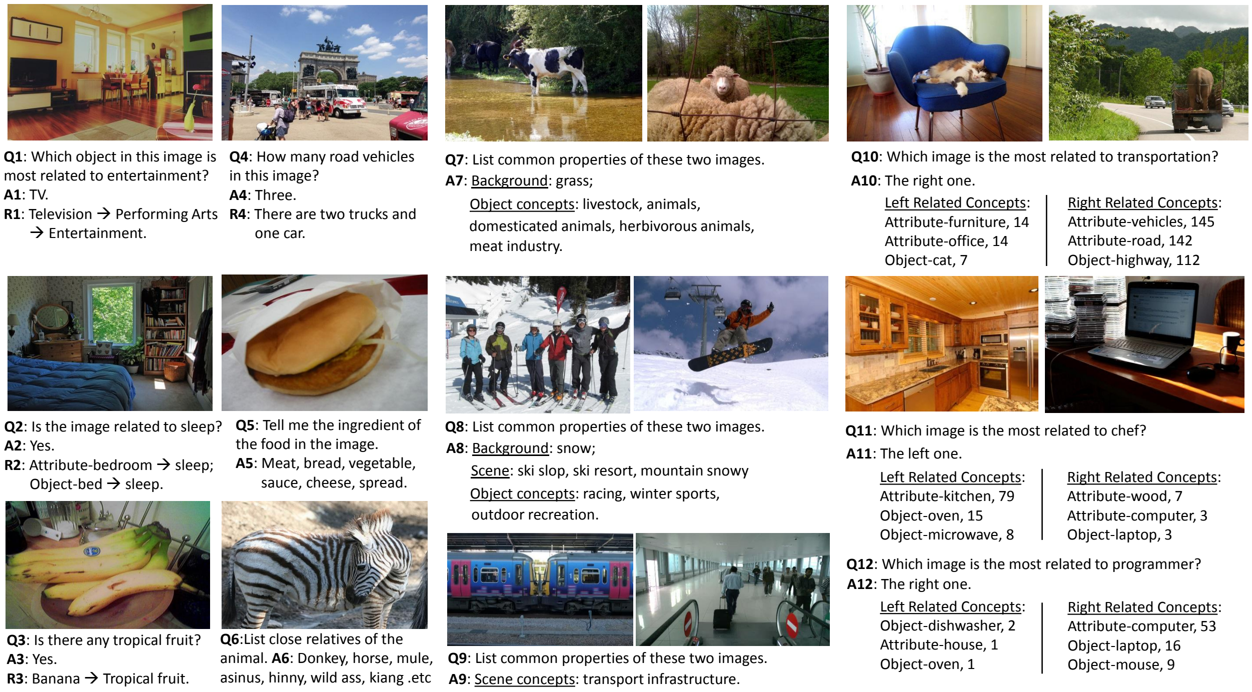

Figure 9: Examples of KB-VQA questions and the answers and reasons given by Ahab. Q1-Q6 are questions involving single images and Q7-Q12 are questions involving two images. The numbers after visual concepts in Q10-Q12 are scores measuring the correlation between visual concepts and concepts given in questions (see supplementary material).

their scene categories (railway station and airport) are linked to the same concept "transport infrastructure" in DBpedia. For the second type, the correlation between each visual concept and the query concept is measured by a scoring function (using the same strategy as is used for *IsImgRelate* and *MostRelObj* in Section 3.2), and the correlation between an image and this concept is calculated by averaging the top three scores. As we can see in Q11 and Q12, attributes "kitchen" and "computer" are most related to the concepts "chef" and "programmer" respectively, so it is easy to judge that the answer for Q11 is the left image and the one for Q12 is the right.

The flexibility of Quepy, and the power of Python, make adding additional question types quite simple. It would be straightforward to add question types requiring an image as an answer, for instance (IMG1+NLQ $\rightarrow$ IMGs).

5 Conclusion

We have described a method capable of reasoning about the content of general images, and interactively answering a wide variety of questions about them. The method develops a structured representation of the content of the image, and relevant information about the rest of the world, on the basis of a large external knowledge base. It is capable of explaining its reasoning in terms of the entities in the knowledge base, and the connections between them. Ahab is applicable to any knowledge base for which a SPARQL interface is available. This includes any of the over a thousand RDF datasets online [35] which relate information on taxonomy, music, UK government statistics, Brazilian politicians, and the articles of the New York Times, amongst a host of other topics. Each could be used to provide a specific visual question answering capability, but many can also be linked by common identifiers to form larger repositories. If a knowledge base containing common sense

were available, the method we have described could use it to draw sensible general conclusions about the content of images.

We have also provided a dataset and methodology for testing the performance of general visual question answering techniques, and shown that Ahab substantially outperforms the currently predominant visual question answering approach when so tested.

References

[1] S. Antol, A. Agrawal, J. Lu, M. Mitchell, D. Batra, C. L. Zitnick, and D. Parikh. VQA: Visual Question Answering - Version 2. *arXiv:1505.00468v2*, 2015.

[2] S. Auer, C. Bizer, G. Kobilarov, J. Lehmann, R. Cyganiak, and Z. Ives. *DBpedia: A nucleus for a web of open data*. Springer, 2007.

[3] M. Banko, M. J. Cafarella, S. Soderland, M. Broadhead, and O. Etzioni. Open information extraction for the web. In *Proc. Int. Joint Conf. Artificial Intelligence*, 2007.

[4] J. Berant, A. Chou, R. Frostig, and P. Liang. Semantic Parsing on Freebase from Question-Answer Pairs. In *Proc. Empirical Methods in Natural Language Processing*, pages 1533–1544, 2013.

[5] S. Bird, E. Klein, and E. Loper. *Natural language processing with Python*. O'Reilly Media, Inc., 2009.

[6] K. Bollacker, C. Evans, P. Paritosh, T. Sturge, and J. Taylor. Freebase: a collaboratively created graph database for structuring human knowledge. In *Proc. ACM SIGMOD/PODS Conf.*, pages 1247–1250, 2008.

[7] A. Bordes, S. Chopra, and J. Weston. Question answering with subgraph embeddings. *arXiv:1406.3676*, 2014.

[8] Q. Cai and A. Yates. Large-scale Semantic Parsing via Schema Matching and Lexicon Extension. In *Proc. Conf. the Association for Computational Linguistics*, 2013.

[9] A. Carlson, J. Betteridge, B. Kisiel, and B. Settles. Toward an Architecture for Never-Ending Language Learning. In *Proc. AAAI Conf. Artificial Intelligence*, 2010.

[10] X. Chen, A. Shrivastava, and A. Gupta. Neil: Extracting visual knowledge from web data. In *Proc. Int. Conf. Computer Vision*, 2013.

[11] X. Chen, A. Shrivastava, and A. Gupta. Enriching visual knowledge bases via object discovery and segmentation. In *Proc. IEEE Conf. Computer Vision Pattern Recognition*, 2014.

[12] J. Deng, W. Dong, R. Socher, L.-J. Li, K. Li, and L. Fei-Fei. Imagenet: A large-scale hierarchical image database. In *Proc. IEEE Conf. Computer Vision and Pattern Recognition*, 2009.

[13] O. Erling. Virtuoso, a Hybrid RDBMS/Graph Column Store. *IEEE Data Eng. Bull.*, 35(1):3–8, 2012.

[14] O. Etzioni, A. Fader, J. Christensen, S. Soderland, and M. Mausam. Open Information Extraction: The Second Generation. In *Proc. Int. Joint Conf. Artificial Intelligence*, 2011.

[15] A. Fader, S. Soderland, and O. Etzioni. Identifying relations for open information extraction. In *Proc. Empirical Methods in Natural Language Processing*, 2011.

[16] A. Fader, L. Zettlemoyer, and O. Etzioni. Open question answering over curated and extracted knowledge bases. In *Proc. ACM SIGKDD Conference on Knowledge Discovery and Data Mining*, 2014.

[17] H. Gao, J. Mao, J. Zhou, Z. Huang, L. Wang, and W. Xu. Are You Talking to a Machine? Dataset and Methods for Multilingual Image Question Answering. In *Proc. Int. Conf. Adv. Neural Information Processing Systems*, 2015.

[18] D. Geman, S. Geman, N. Hallonquist, and L. Younes. Visual Turing test for computer vision systems. *Proceedings of the National Academy of Sciences*, 112(12):3618–3623, 2015.

[19] R. Girshick. Fast R-CNN. *arXiv:1504.08083*, 2015.

[20] R. W. Group et al. Resource description framework, 2014. `http://www.w3.org/standards/techs/rdf`.

[21] S. Hochreiter and J. Schmidhuber. Long short-term memory. *Neural computation*, 9(8):1735–1780, 1997.

[22] J. Hoffart, F. M. Suchanek, K. Berberich, and G. Weikum. YAGO2: A spatially and temporally enhanced knowledge base from Wikipedia. In *Proc. Int. Joint Conf. Artificial Intelligence*, 2013.

[23] O. Kolomiyets and M.-F. Moens. A survey on question answering technology from an information retrieval perspective. *Information Sciences*, 181(24):5412–5434, 2011.

[24] A. Krizhevsky, I. Sutskever, and G. E. Hinton. Imagenet classification with deep convolutional neural networks. In *Proc. Int. Conf. Adv. Neural Information Processing Systems*, 2012.

[25] T. Kwiatkowski, E. Choi, Y. Artzi, and L. Zettlemoyer. Scaling semantic parsers with on-the-fly on-

tology matching. In *Proc. Empirical Methods in Natural Language Processing*, 2013.

[26] P. Liang, M. I. Jordan, and D. Klein. Learning dependency-based compositional semantics. *Computational Linguistics*, 39(2):389–446, 2013.

[27] T.-Y. Lin, M. Maire, S. Belongie, J. Hays, P. Perona, D. Ramanan, P. Dollár, and C. L. Zitnick. Microsoft COCO: Common objects in context. In *Proc. Eur. Conf. Computer Vision*, 2014.

[28] F. Mahdisoltani, J. Biega, and F. Suchanek. YAGO3: A knowledge base from multilingual Wikipedias. In *CIDR*, 2015.

[29] M. Malinowski and M. Fritz. A multi-world approach to question answering about real-world scenes based on uncertain input. In *Proc. Int. Conf. Adv. Neural Information Processing Systems*, pages 1682–1690, 2014.

[30] M. Malinowski and M. Fritz. Towards a Visual Turing Challenge. *arXiv:1410.8027*, 2014.

[31] M. Malinowski, M. Rohrbach, and M. Fritz. Ask Your Neurons: A Neural-based Approach to Answering Questions about Images. In *Proc. Int. Conf. Computer Vision*, 2015.

[32] E. Prud'Hommeaux, A. Seaborne, et al. SPARQL query language for RDF. *W3C recommendation*, 15, 2008.

[33] M. Ren, R. Kiros, and R. Zemel. Image Question Answering: A Visual Semantic Embedding Model and a New Dataset. In *Proc. Int. Conf. Adv. Neural Information Processing Systems*, 2015.

[34] T. Rocktäschel, E. Grefenstette, K. M. Hermann, T. Kočiskỳ, and P. Blunsom. Reasoning about Entailment with Neural Attention. *arXiv:1509.06664*, 2015.

[35] M. Schmachtenberg, C. Bizer, and H. Paulheim. State of the LOD Cloud 2014, 2014. `http://linkeddatacatalog.dws.informatik.unimannheim.de/state`.

[36] K. Simonyan and A. Zisserman. Very deep convolutional networks for large-scale image recognition. *arXiv:1409.1556*, 2014.

[37] C. Szegedy, W. Liu, Y. Jia, P. Sermanet, S. Reed, D. Anguelov, D. Erhan, V. Vanhoucke, and A. Rabinovich. Going deeper with convolutions. In *Proc. IEEE Conf. Computer Vision and Pattern Recognition*, 2015.

[38] C. Unger, L. Bühmann, J. Lehmann, A.-C. Ngonga Ngomo, D. Gerber, and P. Cimiano. Template-based question answering over RDF data. In *WWW*, 2012.

[39] D. Vrandečić and M. Krötzsch. Wikidata: a free collaborative knowledgebase. *Communications of the ACM*, 57(10):78–85, 2014.

[40] Q. Wu, C. Shen, A. van den Hengel, L. Liu, and A. Dick. Image Captioning with an Intermediate Attributes Layer. *arXiv:1506.01144v2*, 2015.

[41] X. Yao and B. Van Durme. Information extraction over structured data: Question answering with Freebase. In *Proc. Annual Meeting of the Association for Computational Linguistics*, 2014.

[42] B. Zhou, A. Lapedriza, J. Xiao, A. Torralba, and A. Oliva. Learning deep features for scene recognition using places database. In *Proc. Int. Conf. Adv. Neural Information Processing Systems*, 2014.

[43] Y. Zhu, C. Zhang, C. Ré, and L. Fei-Fei. Building a large-scale multimodal Knowledge Base for Answering Visual Queries. *arXiv:1507.05670*, 2015.

A Mapping ⟨*concept*⟩ Phrases to KB-Entities with Redirections

Given a natural language phrase representing a real-world or abstract concept, we simply search its corresponding KB-entities by matching the phrase to the name of KB-entities. The following SPARQL query is used in Ahab to search the entities in DBpedia corresponding to "Religion":

```
SELECT DISTINCT ?x WHERE {
  ?x label "Religion"@en.
}
```

where the name of an entity is obtained through the predicate `label` (see Table 3). The output of the above query is:

```
http://dbpedia.org/resource/Category:Religion
http://dbpedia.org/resource/Religion
```

which shows that there are two KB entities matched to "Religion". The first one is a category entity and the second one is a non-category entity (see Section 3.2 of the main body).

Note that in natrual language, the same concept can be expressed in many ways, which can be caused by synonyms, capitalizations, punctuation, tenses, abbreviation and even misspellings. For example, both "Frisbee" and "Flying disc" correspond to the disc-shaped gliding toy; and the American technology company producing iPhone can be expressed as "Apple Company" and "Apple Inc.".

To handle this issue, DBpedia introduces a number of "dummy" entities that are only used to point to a "concrete" entity, such that all different expressions of a concept are redirected to one concrete entity.

If the input phrase is the abbreviation "Relig." rather than "Religion", the following SPARQL query can still locate the KB entity `KB:Religion`:

```
SELECT DISTINCT ?x1 WHERE {
  ?x0 label "Relig."@en.
  ?x0 wikiPageRedirects ?x1.
}
```

which outputs

```
http://dbpedia.org/resource/Religion
```

By using redirections, the vocabulary of concept phrases is enriched significantly.

B Query Generation and Post-processing

Given all the slot-phrases mapped to KB entities (see Section 3.2 in the main body), the next step is to generate queries specific to question templates, with post-processing steps for some templates.

The SPARQL queries used to answer specific questions are shown in the following. There are two types of SPARQL queries (see [32] for details): `ASK` queries check whether or not a query pattern has a solution, which returns 1 if there is at least one solution and returns 0 otherwise; `SELECT` queries returns variables of all matched solutions. In the following queries, the terms start with `?` correspond to variables and others correspond to fixed entities or predicates. Table 3 shows the definition of the involved entities and predicates.

WhatIs The short abstract of the Wikipedia page corresponding to `KB:obj` is returned.

```
SELECT ?desc WHERE {
  KB:obj comment ?desc.
}
```

ColorOf The color of the mapped object is returned using the following query.

```
SELECT DISTINCT ?obj_color {
  Obj color ?obj_color.
}
```

IsSameThing If the mapped two objects correspond to the same category name, the following query returns true; otherwise, it returns false.

```
ASK {
  KB:obj1 name ?obj_nm.
  KB:obj2 name ?obj_nm.
}
```

ListObj The category names of all objects contained in the questioned image are returned via the following query.

```
SELECT DISTINCT ?obj_nm {
  Img contain ?obj.
  ?obj name ?obj_nm.
}
```

ImgScene The scene information can be obtained from image attributes or scenes, using the following queries. As attributes are trained with COCO data, they have higher priorities.

```
SELECT DISTINCT ?att_nm {
  Img  img-att ?att.
  ?att supercat-name "scene".
  ?att name ?att_nm.
}

SELECT DISTINCT ?scn_nm {
  Img  img-scn ?scn.
  ?scn name ?scn_nm.
}
```

ObjAction The following query returns the action attributes of an image.

```
SELECT DISTINCT ?att_name {
  Img  img-att ?att.
  ?att supercat-name "action".
}
```

IsThereAny*, *IsTheA*, *AreAllThe*, *LargestObj* and *HowMany The following query checks whether or not `KB:concept` is a transitive category of `KB:obj`, which is used by the routines for the above five question types to determine if there is a hyponymy relationship between `KB:concept` and `KB:obj`.

```
ASK {
  KB:obj subject/broader?/broader? KB:concept.
}
```

where a transitive closure is used (see [32] for details) to find all transitive categories of `KB:obj` within three steps. In this work, all transitive categories of an entity is limited to three steps, to avoid arriving at too general concepts.

For *IsThereAny*: true is returned if at least one object in the questioned image passes the above query (query returns 1); otherwise, false is returned.

For *IsTheA*: if the identified object passes the query, return true; otherwise, return false.

For *AreAllThe*: return true, only if all identified objects pass the query.

For *LargestObj*: from all passed objects, return the one with the largest size.

For *HowMany*: return the number of passed objects.

IsImgRelate* and *MostRelObj For *MostRelObj*, the correlation of an object `KB:obj` and a concept `KB:concept` is measured by the function

$$f = \alpha \cdot f_1 + f_2, \quad (1)$$

where f_1 and f_2 are the respective outputs of the following two queries; and α is the weight of f_1.

```
ASK {
  { KB:obj WikiLink KB:concept } UNION
  { KB:concept WikiLink KB:obj } UNION
  { KB:obj subject/broader?/broader? KB:concept }.
}
SELECT COUNT(DISTINCT ?x) WHERE {
  { KB:obj WikiLink ?x } UNION
  { ?x WikiLink KB:obj }.
  { KB:concept WikiLink ?x } UNION
  { ?x WikiLink KB:concept }.
}
```

The first query returns 1 if `KB:concept` is a transitive category of `KB:obj`, or they are directly linked by predicate `WikiLink`. The second query counts the number of indirect links via `WikiLink` and another KB-entity `?x`. Explicitly, the importance of f_1 is greater than f_2, so α is set to 50 in the experiments.

For question *IsImgRelate*, the correlation of the concept and each object/attribute/scene of the questioned image is measured by the same scoring function (1). If any score is larger than 50, the image is considered as being related to the concept.

This scoring function is used for question "Which image is the most related to ⟨*concept*⟩?" as well.

CommProp The transitive categories shared by both `KB:obj1` and `KB:obj2` are returned through:

```
SELECT DISTINCT ?cat WHERE {
  KB:obj1 subject/broader?/broader? ?cat.
  KB:obj2 subject/broader?/broader? ?cat.
}
```

Note that `KB:obj2` can be replaced by `KB:concept` based on the asked question.

For question "List common properties of these two images.", the common transitive categories are obtained by comparing the objects/attributes/scenes of two images.

SportEquip In DBpedia, the equipments for a specific sport share a common KB-category. This KB-category has the label "⟨sport-name⟩ equipment", where ⟨sport-name⟩ is the name of a sport (such as "Tennis", "Skiing"). The sport name can be obtained from image attributes. The following query returns all tennis equipments:

```
SELECT DISTINCT ?equip WHERE {
  ?equip subject ?cat.
```

```
  ?cat   label ?cat_nm.
  FILTER regex(?cat_nm, "Tennis equipment").
}
```

which uses a regular expression (regex) to parse the label of KB-categories.

If the sport information is not contained in attributes. We can answer this question by the following query, which returns all equipments sharing the same "⟨sport-name⟩ equipment"-category with a detected object in the image.

```
SELECT DISTINCT ?equip WHERE {
  Img    contain ?obj.
  ?obj    subject ?cat.
  ?equip subject ?cat.
  ?cat   broader/broader? KB:Cat-Sports_equipment.
}
```

where the entity KB:Cat-Sports_equipment[8] is a super-category of all "⟨sport-name⟩ equipment"-categories.

LocIntro The place where a KB-entity is invented is recorded by a KB-category with label "⟨country⟩ inventions".

```
SELECT DISTINCT ?cat_nm WHERE {
  KB:obj subject ?cat.
  ?cat   label ?cat_nm.
  FILTER regex(?cat_nm,"^[a-z|A-z]+ inventions$").
}
```

YearIntro*, *FirstIntro* and *ListSameYear The introduction year of a KB-entity is recorded by a KB-category with label "⟨year⟩ introductions". The following query returns the introduction year of KB:obj, which is used for question type *YearIntro*:

```
SELECT DISTINCT ?cat_nm WHERE {
  KB:obj subject ?cat.
  ?cat   label ?cat_nm.
  FILTER regex(?cat_nm,"^[0-9]+ introductions$").
}
```

For *FirstIntro*, the introduction years of two entities are compared.

For *ListSameYear*, the following query returns all things introduced in the same year of KB:obj:

```
SELECT DISTINCT ?thing_nm WHERE {
  ?thing subject ?cat.
  ?thing label ?thing_nm.
  KB:obj subject ?cat.
  ?cat   label ?cat_nm.
  FILTER regex(?cat_nm,"^[0-9]+ introductions$").
}
```

FoodIngredient With predicate ingredient, the following query returns the ingredients of KB:food.

```
SELECT DISTINCT ?Ingrd_nm WHERE {
  KB:food ingredient ?Ingrd.
  ?Ingrd  label      ?Ingrd_nm.
}
```

AnimalClass*, *AnimalRelative* and *AnimalSame With predicate taxonomy (see Table 3), the following queries are used for the above three templates respectively.

```
SELECT DISTINCT ?class_nm WHERE {
  KB:animal taxonomy ?class.
  ?class    label    ?class_nm.
}
SELECT DISTINCT ?relative_nm WHERE {
  KB:animal taxonomy ?class.
  ?relative taxonomy ?class.
  ?relative label    ?relative_nm.
}
ASK {
  KB:animal1 taxonomy ?class.
  KB:animal2 taxonomy ?class.
}
```

C Evaluation Protocol

A user-friendly interface (in Matlab) is developed for the human subject (examiner) evaluation. During the time of evaluation, a question, an image and three answers are popped up. Those answers are generated by LSTM, our system and the other human subject, respectively (see Figure 10). Our evaluation process is double-blind, that means, examiners are different from those questioners (who ask questions). Moreover, the answer sources (*i.e.*, which algorithm generated the answer) are not provided to the examiner.

We ask the examiners to give a correctness score (1-5) for each answer as following:

1: Totally wrong (if the examiner thinks the candidate answer is largely different from it suppose to be or even wrong answer type).

[8] The URI is http://dbpedia.org/resource/Category:Sports_equipment

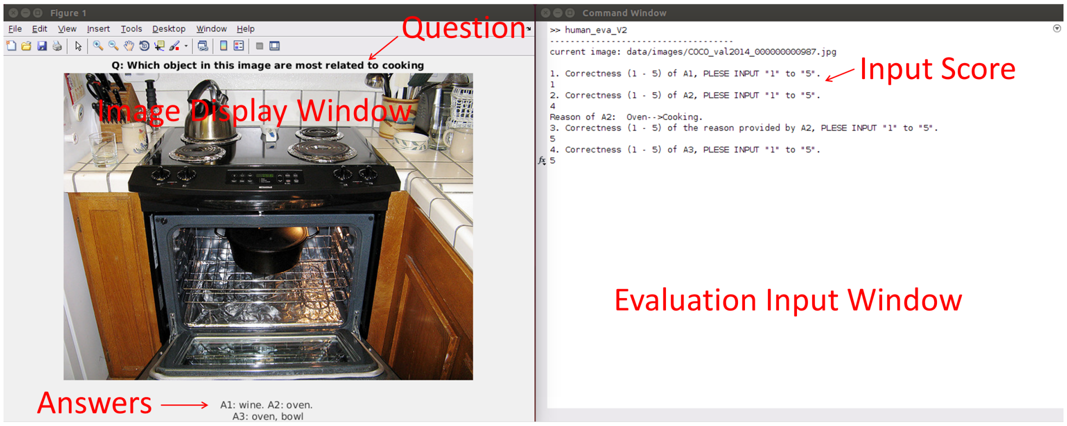

Figure 10: Our evaluation tool interface, written in Matlab. The left side is the image, question and answers displaying side. The right side is the user input side. Answers are labelled as A1, A2 and A3, so the examiner has no clue about the source of the answers. During the evaluation, users (examiners) are only required to input a score from 1 to 5 for each evaluation item.

2: Slightly wrong (if the examiner thinks the candidate answer is wrong, but close to right. For example, the groundtruth answer is "pink" while the generated answer is "red").

3: Borderline (applicable when the groundtruth answer is a list. if the examiner thinks the candidate answers only hit little amount of the answers in the list).

4: OK (applicable when the groundtruth answer is a list. if the examiner thinks the candidate answers hit most of the answers in the list).

5: Perfect (if the examiner thinks the candidate answer is perfectly correct).

Finally, the overall accuracy for a given question type is calculated by $\frac{\#\text{ of answers that correctness}>3}{\text{Total }\#\text{ of questions}}$. The same rule is applied for the "logical reason" correctness evaluation.

D Visual Concepts

Three types of visual concepts are detected in Ahab, which are object, attributes and scenes. The scene classes are defined the same as in the MIT Places205 dataset [42]. The object classes are obtained by merging the 80 classes in MS COCO object dataset [27] and 200 classes in ImageNet object dataset [12], which are shown in Table 5. The vocabulary of attributes trained in [40] is demonstrated in Table 4.

E Examples

Examples of KB-VQA questions and the answers generated by Ahab are shown in Fig. 11.

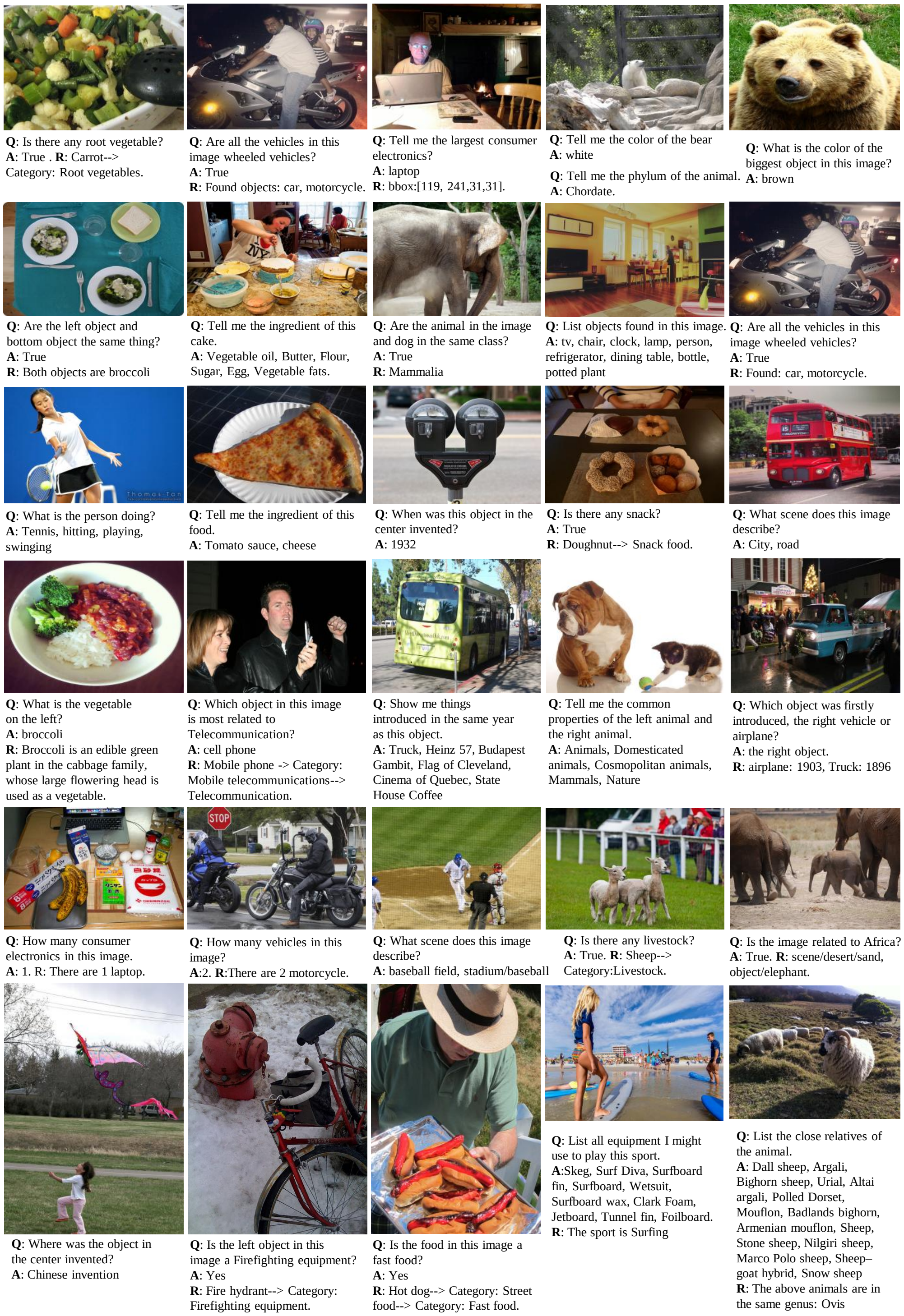

Figure 11: Examples of KB-VQA questions and the answers generated by Ahab. **Q**: questions; **A**: answers; **R**: reasons.

Term	Defined by	Description
**Entities**		
`Img`	Ahab	The Ahab entity corresponding to the questioned image.
`Obj`	Ahab	The Ahab entity mapped to slot ⟨*obj*⟩.
`KB:obj`	DBpedia	The KB-entity mapped to slot ⟨*obj*⟩.
`KB:concept`	DBpedia	The KB-entity mapped to slot ⟨*concept*⟩.
`KB:food`	DBpedia	The KB-entity mapped to slot ⟨*food*⟩.
`KB:animal`	DBpedia	The KB-entity mapped to slot ⟨*animal*⟩.
**Predicates**		
`img-att`	Ahab	Linking an image to one of its attribute categories.
`img-scn`	Ahab	Linking an image to one of its scene categories.
`contain`	Ahab	Linking an image to one of the objects it contains.
`name`	Ahab	Linking an object/scene/attribute category to its name (string).
`color`	Ahab	Linking an object to its color.
`size`	Ahab	Linking an object to its size.
`supercat-name`	Ahab	Linking an object/scene/attribute category to its super-category name (string).
`label`	DBpedia	Linking a KB-entity to its name (string).<br>Its URI is `http://www.w3.org/2000/01/rdf-schema#label`.
`comment`	DBpedia	Linking a KB-entity to its short description.<br>Its URI is `http://www.w3.org/2000/01/rdf-schema#comment`.
`wikiPageRedirects`	DBpedia	Linking a "dummy" KB-entity to the "concrete" one describing the same concept.<br>Its URI is `http://dbpedia.org/ontology/wikiPageRedirects`
`subject`	DBpedia	Linking a non-category KB-entity to its categories.<br>Its URI is `http://purl.org/dc/terms/subject`.
`broader`	DBpedia	Linking a category KB-entity to its super-categories.<br>Its URI is `http://www.w3.org/2004/02/skos/core#broader`.
`Wikilink`	DBpedia	Linking two correlated non-category KB-entities, which is extracted from the internal links bettween Wikipedia articles.<br>Its URI is `http://dbpedia.org/ontology/wikiPageWikiLink`.
`ingredient`	DBpedia	Linking a food KB-entity to its ingredient, which is extracted from infoboxes of Wikipedia.<br>Its URI is `http://dbpedia.org/ontology/ingredient`.
`taxonomy`	DBpedia	Linking an animal KB-entity to its taxonomy class (can be kingdom, phylum, class, order, family or genus), which is extracted from infoboxes of Wikipedia.<br>For phylum, the URI is `http://dbpedia.org/ontology/phylum`.

Table 3: The RDF entities and predicates used in Ahab, some of which are defined by Ahab and others are originally defined by DBpedia.

Super-category	Number	Attribute categories
Action	24	playing, sitting, standing, swinging, catching, cutting, dining, driving, eating, flying, hitting, jumping, laying, racing, reads, swimming, running, sleeping, smiling, taking, talking, walking, wearing, wedding
Sport	4	surfing, tennis, baseball, skateboard
Scene	24	mountain, road, snow, airport, bathroom, beach, bedroom, city, court, forest, hill, island, kitchen, lake, market, ocean, office, park, river, room, sea, sky, restaurant, field zoo
Object	95	children, bottle, computer, drink, glass, monitor, tree, wood, basket, bathtub, beer, blanket, box, bread, bridge, buildings, cabinets, camera, candles, cheese, chicken, chocolate, church, clouds, coat, coffee, decker, desk, dishes, door, face, fence, fish, flag, flowers, foods, fruits, furniture, grass, hair, hands, head, helmet, hotdog, house, ice, jacket, kitten, lettuce, lights, luggage, meat, metal, mouth, onions, palm, pants, papers, pen, pillows, plants, plates, players, police, potatoes, racquet, railing, rain, rocks, salad, sand, seat, shelf, ship, shirt, shorts, shower, sofa, station, stone, suit, sunglasses, toddler, tomatoes, towel, tower, toys, tracks, vegetables, vehicles, wall, water, wii, windows, wine

Table 4: The 147 image atributes used as visual concepts in Ahab system.

Super-category	Number	Object categories
person	1	person
vehicle	12	bicycle, car, motorcycle, airplane, bus, train, truck, boat, cart, snowmobile, snowplow, unicycle
outdoor	5	traffic light, fire hydrant, stop sign, parking meter, bench
animal	48	cat, dog, horse, sheep, cow, elephant, bear, zebra, giraffe, ant, antelope, armadillo, bee, butterfly, camel, centipede,dragonfly, fox, frog, giant panda, goldfish, hamster, hippopotamus, isopod, jellyfish, koala bear, ladybug, lion, lizard, lobster,monkey, otter, porcupine, rabbit, ray, red panda, scorpion, seal, skunk, snail, snake, squirrel, starfish, swine, tick, tiger, turtle,whale
accessory	18	backpack, umbrella, handbag, tie, suitcase, band aid, bathing cap, crutch, diaper, face powder, hat with awide brim, helmet, maillot, miniskirt, neck brace, plastic bag, stethoscope, swimming trunks
sports	22	frisbee, skis, snowboard, kite, baseball bat, baseball glove, skateboard, surfboard, tennis racket, balance beam, baseball,basketball, croquet ball, golf ball, golfcart, horizontal bar, punching bag, racket, rugby ball,soccer ball, tennis ball, volleyball
kitchen	20	bottle, wine glass, cup, fork, knife, spoon, bowl, beaker, can opener, cocktail shaker, corkscrew, frying pan, ladle, milk can,pitcher, plate rack, salt or pepper shaker, spatula, strainer, water bottle
food	27	banana, apple, sandwich, orange, broccoli, carrot, hot dog, pizza, donut, cake, artichoke, bagel, bell pepper, burrito, cream,cucumber, fig, guacamole, hamburger, head cabbage, lemon, mushroom, pineapple, pomegranate, popsicle, pretzel,strawberry
furniture	8	chair, couch, potted plant, bed, dining table, toilet, baby bed, filing cabinet
electronic	7	tv, laptop, mouse, remote, keyboard, cell phone, iPod
appliance	14	microwave, oven, toaster, sink, refrigerator, coffee maker, dishwasher, electric fan, printer, stove, tape player, vacuum,waffle iron, washer
indoor	18	clock, vase, scissors, teddy bear, hair drier, toothbrush, binder, bookshelf, digital clock, hair spray, lamp, lipstick, pencil box, pencil sharpener, perfume, rubber eraser, ruler, soap dispenser
music	16	accordion, banjo, cello, chime, drum, flute, french horn, guitar, harmonica, harp, oboe, piano, saxophone,trombone, trumpet, violin
tool	8	axe, bow, chain saw, hammer, power drill, screwdriver, stretcher, syringe

Table 5: The 224 object classes used as visual concepts in Ahab system.
